# Genome-wide analysis of DNA methylation in photoperiod- and thermo-sensitive male sterile rice Peiai 64S

**DOI:** 10.1186/s12864-015-1317-7

**Published:** 2015-02-19

**Authors:** Jihong Hu, Xiaojun Chen, Hongyuan Zhang, Yi Ding

**Affiliations:** State Key Laboratory of Hybrid Rice, College of Life Sciences, Wuhan University, Wuhan, 430072 People’s Republic of China

**Keywords:** *Oryza sativa*, DNA methylation, PTGMS, Fertility transition, Gene expression

## Abstract

**Background:**

Epigenetic modifications play important roles in the regulation of plant development. DNA methylation is an important epigenetic modification that dynamically regulates gene expression during developmental processes. However, little studies have been reported about the methylation profiles of photoperiod- and thermo-sensitive genic male sterile (PTGMS) rice during the fertility transition.

**Results:**

In this study, using methylated DNA immunoprecipitation sequencing (MeDIP-seq), the global DNA methylation patterns were compared in the rice PTGMS line PA64S under two different environments (different temperatures and day lengths). The profiling of the DNA methylation under two different phenotypes (sterility and fertility) revealed that hypermethylation was observed in PA64S (sterility), and 1258 differentially methylated regions (DMRs) were found between PA64S (sterility) and PA64S (fertility). Twenty differentially methylated genes of them were further validated through bisulfite sequencing, and four of these genes were analyzed by qRT-PCR. Especially, a differentially methylated gene (LOC_Os08g38210), which encoded transcription factor *BIM2*, is a component of brassinosteroid signaling in rice. The hypermethylated *BIM2* gene may suppress some downstream genes in brassinosteroid signaling pathway, and thus affect the male fertility in PA64S.

**Conclusions:**

The results presented here indicated that hypermethylation was observed in PA64S (sterility). Gene Ontology (GO) analysis and KEGG analysis revealed that flavone and flavonol biosynthrsis, circadian rhythm, photosynthesis and oxidative phosphorylation pathways were involved in sterility-fertility transition of PA64S.

**Electronic supplementary material:**

The online version of this article (doi:10.1186/s12864-015-1317-7) contains supplementary material, which is available to authorized users.

## Background

DNA methylation is an indispensable epigenetic modification of plant genomes. It occurs predominantly in the CG context in both plants and animals [1-3]. The state of CpG methylation regulates and stabilizes chromatin structure, and possibly regulates the accessibility of these DNA regions to the transcriptional machinery [4]. Studies have shown that DNA methylation plays an important role in many plant processes, including transposon silencing, virus defence, and gene imprinting [5-7].

In plant, DNA methylation is mainly distributed in heterochromatic regions that are composed of tandem or inverted repeats, i.e., transposons. Transposable elements (TEs) are usually highly methylated along their entire length, but DNA methylation within genes is distributed away from the 5′ and 3′ ends. It has been proposed that DNA methylation is primarily used to inhibit the transcription and movement of TEs to protect genomes against uncontrolled insertions. Some studies have reported that transposons or retrotransposons are activated in response to environmental stress and may generate true genetic or epigenetic changes for adapting to stresses [8]. In rice, epigenetic regulation has been reported to affect the transposition activity of *Tos17* and modulates the activity of the neighboring genes [9]. Highly methylated transposons may affect transcription of neighboring genes. For example, the patterns of DNA methylation in long terminal repeat (LTR) transposable elements differed between rice leaves and roots and affected the transcription of the neighboring genes [10].

Recently, three DNA treatment techniques have been typically employed for DNA methylation detection: (i) methylation sensitive amplified polymorphism (MSAP), (ii) methylated DNA enrichment (MeDIP), (iii) bisulfite-sequencing. With the advent of new technologies, the elucidation of the genome-wide methylation profiles is possible. In this regard, the immunoprecipitation of methylated DNA by monoclonal antibodies specific to 5-methylcytidine (5mC) combined with high-throughput sequencing (MeDIP-seq) has been used as a valuable tool to map methylated DNA at the genomic scale [11,12]. The MeDIP-seq approach employs an antibody against 5-methylcytosine or methyl-binding domain proteins to capture methylated DNA, which are subsequently subjected to next generation sequencing [11]. After methylated DNA enrichment, the unmethylated DNA fragments are removed from the genome samples for reducing the redundancy. Thus, a relatively lower sequencing throughput is required, compared with bisulfite-sequencing. In addition, MeDIP is more unbiased and less limited than MSAP. Due to these advantages, MeDIP-seq is considered to have great potential for the development of cost-effective and unbiased strategies for whole genome DNA methylation profiling.

Rice (*Oryza sativa* L.) is one of the most important grain crops worldwide and provides a staple food for almost half of the world’s population [13]. Recently, hybrid rice has made a tremendous contribution to food security both in China and many other countries which are losing arable land. Hybrid rice technologies are mainly based on two well-known male sterility systems, namely CMS (Cytoplasmic Male Sterile) and EGMS (Environmentally sensitive Genic Male Sterile). Compared with the three-line system (CMS system), the two-line system, is based on the discovery and application of EGMS lines, which serve as both the male sterile lines and maintainer lines under different environmental conditions [14]. PTGMS (Photoperiod- Thermo-Sensitive Genic Male Sterile) is the major type of EGMS germplasm resources and has been widely used for breeding two-line hybrid rice. The PTGMS system is advantageous for broad restoration ability, easy maintenance, and multiplication [15-17]. Thus, the utilization of PTGMS lines is an important approach for the better exploitation of heterosis in rice.

Peiai 64S (PA64S) is one of the most important *indica* rice genic male sterile lines in two-line system. Its paternal line is Peiai64, and its maternal line is Nongken58, which is the first photoperiod- sensitive genic male sterile line discovered by Mingsong Shi in Hubei, China in 1973 [18]. Using PA64S as the maternal line, more than 10 two-line hybrid rice varieties have been developed since 1996 in China. Among these new varieties, LiangYouPei9 (LYP9) is an elite super hybrid rice and increases grain yield by approximately 20% per hectare compared with other hybrid rice lines in the past years [7,19]. The fertility transition of PA64S was controlled by the photoperiod and temperature: the sterility trait exhibited at temperatures higher than 23.5°C during the anther development and long-day (LD) (≥14 h) conditions can suppress the fertility transition. Therefore, under lower temperatures (~21-23°C) and short-day (SD) (<14 h) conditions during anther development, PA64S can transform from male sterility to fertility [20,21].

To date, some studies have reported that a substitution of C-to-G in the male-fertility-associated noncoding RNA *p/tms12-1* in PA64S produced a mutant small RNA, namely osa-smR5864m. This mutated noncoding small RNA gene may lead to PTGMS in PA64S [21]. Furthermore, the transcriptome of the PGMS rice Nongken 58S was significantly suppressed under LD condition and the circadian rhythm pathway had been shown to be involved in the male sterility transition [22]. Another noncoding RNA called LDMAR was also found in Nongken 58S and was required for the fertility transition. A siRNA (Psi–LDMAR) was detected to associate with the DNA methylation pattern of the LDMAR promoter in Nongken 58S. And increasing methylation in the promoter of LDMAR of Nongken 58S also reduced the expression of LDMAR, leading to male sterility [23]. These results suggested that DNA methylation or RNA-dependent DNA methylation (RdDM) might be involved in the regulation of PTGMS.

Brassinosteroids (BRs), a class of steroid hormones, have been reported to control male fertility by regulating the expression of several key genes involved in anther and pollen development, such as *SPL*/*NZZ*, *TDF1*, *AMS*, *AtMYB103* and *MS1* [24,25]. The basic helix-loop-helix (bHLH) protein BIM1 (BES1-interacting Myc-like1) is a brassinosteroid signaling component involved in regulating BR-induced genes in Arabidopsis [26]. BRI1- EMSSUPRESSOR1 (BES1) is shown to interact with BIM1 and together bind to the E-box of a BR-induced gene [27].

In this study, we investigated the global DNA methylation alterations in young panicles of the PTGMS line PA64S under two different environmental conditions using the MedIP-seq method. The aim of this work was to explore the DNA methylation patterns and their influences on gene expression during the transition from sterile to fertile in PA64S. The results will provide some information for a better understanding of the role of DNA methylation in PTGMS rice.

## Results

### Cytological observation of pollen morphology

**Figure 1:**
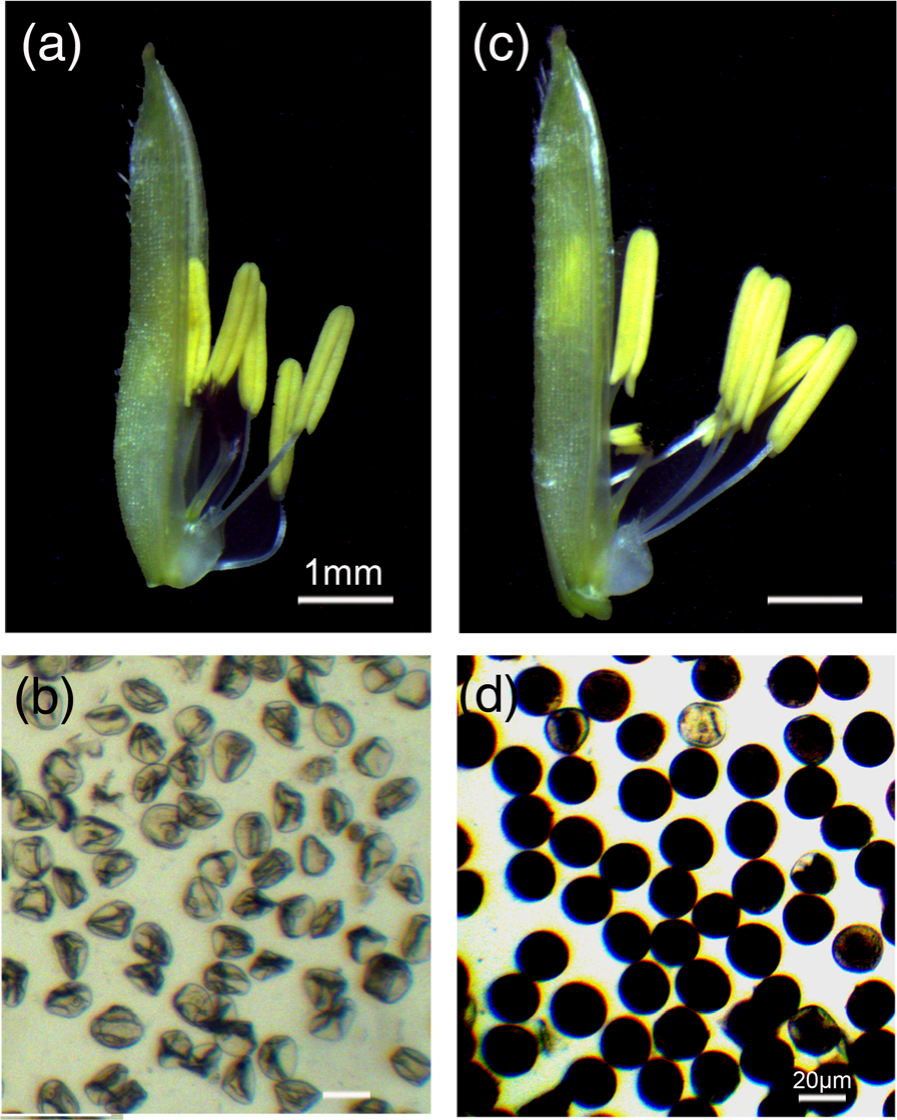
**Cytological observation of pollen morphology. (a)** and **(c)**, the anther phenotypes of PA64S under two environmental conditions. **(b)** and **(d)** 1% I_2_-KI staining of the pollen grains of PA64S under two environmental conditions.

Under higher temperature and LD, anthers from PA64S were light yellow, and its pollen wizened as well as unstained by I_2_-KI (Figure 1a, b), displaying sterility (S). However, under lower temperatures (~21-23°C) and SD condition, PA64S showed male fertility. The anthers of PA64S (fertility, F) were dark yellow, and its spherical pollen could be darkly stained by I_2_-KI, indicating much starch accumulation (Figure 1c, d).

### Genome-wide DNA methylation profiles of PA64S (S) and PA64S (F)

To decipher the genome-wide DNA methylome of PA64S under two different environmental conditions, we immunoprecipitated sheared genomic DNA with an antibody which specifically recognizes 5-methylcytosine and sequenced the enriched methylated DNA. Only the uniquely mapped reads were used in the scanning of the methylation peak. A total of 24,489,796 raw reads were generated for the two samples, and more than 92% of the reads were mapped, and about 58% of the reads in each sample were uniquely mapped to the rice genome in each sample (Table 1 and Additional file 1).

**Table 1:** **Data generated by MeDIP-seq in PA64S (S) and PA64S (F)**

	**Total MeDIP-Seq data**	**Percentage of mapped reads in total reads**	**Unique mapped reads**	**Unique mapped bases**	**Percentage of unique mapped reads**
PA64S(S)	24,489,796	92.05%	14,676,561	719,151,489	59.93%
PA64S(F)	24,489,796	92.20%	14,410,405	706,109,845	58.84%

**Figure 2:**
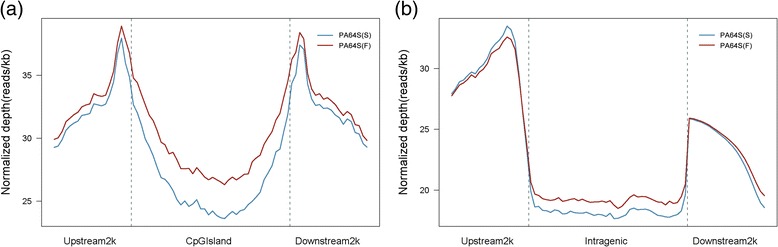
**Distribution of reads around CpG Islands and gene body. (a)** Distribution of reads around CpG Islands; **(b)** Distribution of reads around gene body. The upstream and downstream 2 kb regions were split into 20 equal regions. In the gene body, each gene was split into 40 equal regions. For each region, the normalized number of reads was calculated. The “Y” axis is the average of the normalized depth for each region.

**Figure 3:**
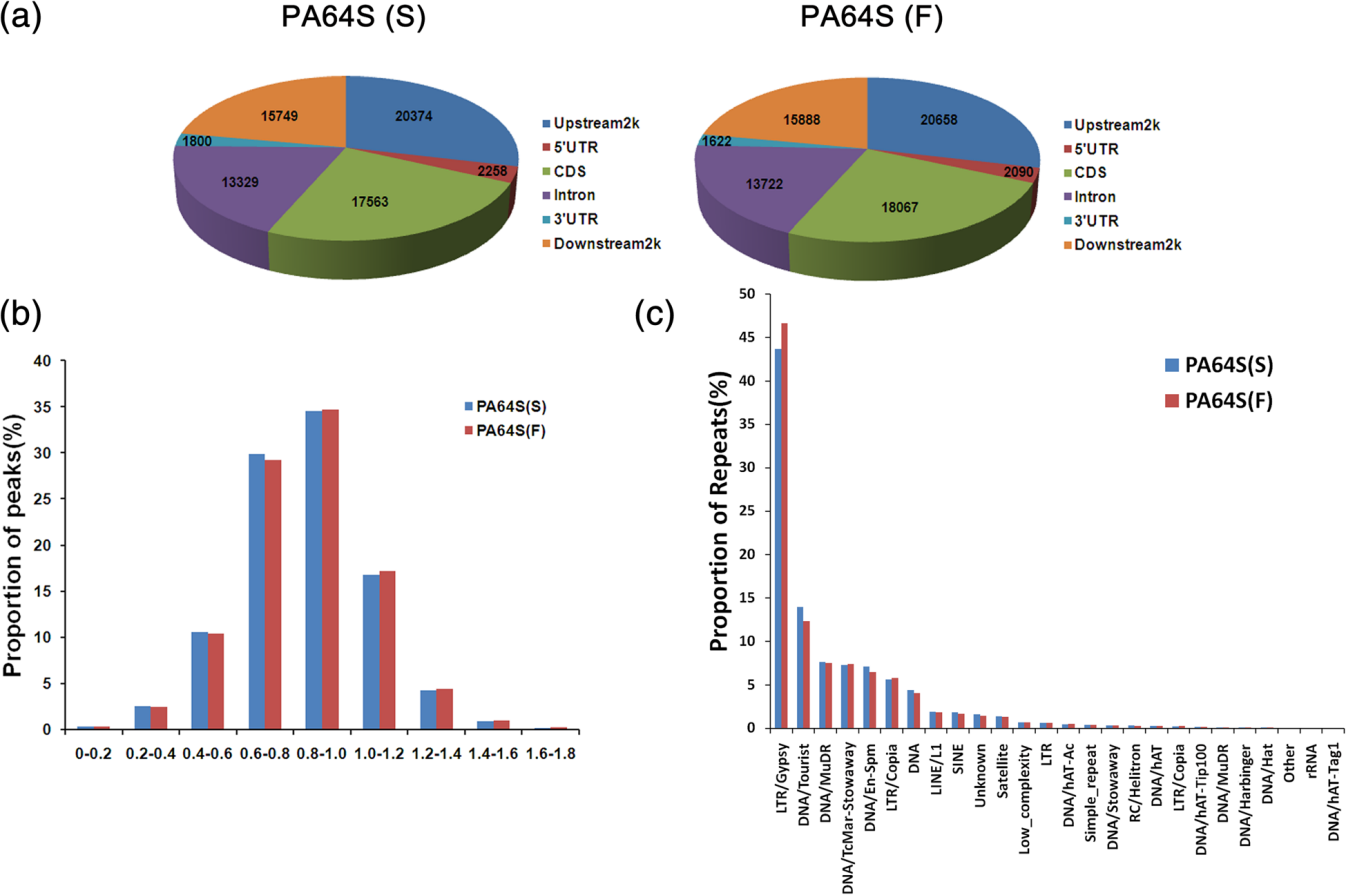
**Different distributions of PA64S(S) and PA64S (F). (a)** Peak distribution in different components of the PA64S(S) and PA64S (F) genome. **(b)** Distribution of methylated peaks of different repeat types in PA64S (S) and PA64S (F). **(c)** Distribution of CpG (O/E) in PA64S(S) and PA64S (F).

According the principle of MedIP method, analysis of the methylation enrichment in different components of the genome showed that 2 kb region upstream and 2 kb region downstream of the CpG Island and intragenic had enriched more methylation reads, especially, when departing from the CpG Island or intragenic regions (Figure 2). Furthermore, the upstream 2k and 5’UTR or downstream 2k and 3’UTR were enriched more reads (Figure 3a).

**Figure 4:**
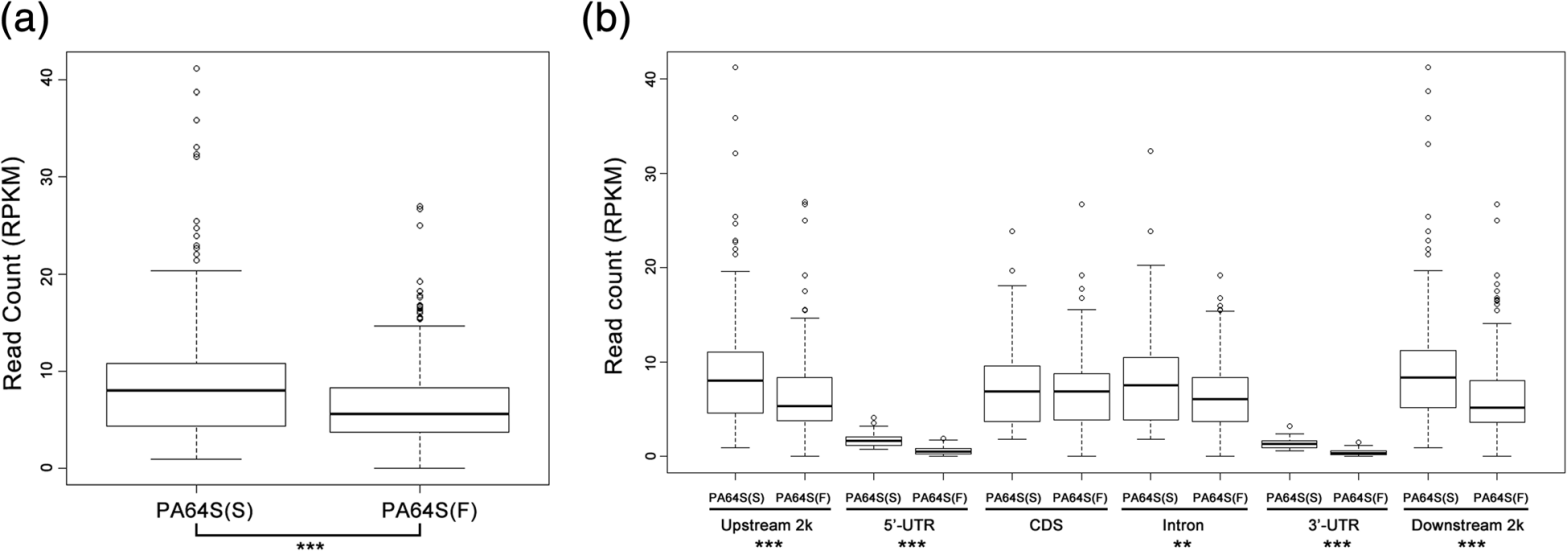
**Boxplots showing the 5mC content (read count, y axis) in 1 kb tiled windows for PA64S(S) and PA64S (F). (a)** Total reads of PA64S(S) and PA64S (F); **(b)** Reads for each elements of PA64S(S) and PA64S (F). The asterisks indicate significant differences between PA64S(S) and PA64S (F), as determined by Student’s *t* test (**P < 0.01, ***P < 0.001).

The CpG observed/expected (O/E) ratio of PA64S (S) and PA64S (F) was mainly in a range of 0.4 to 1.2. There was no significant difference in the CpG (O/E) ratio between PA64S(S) and PA64S (F). However, PA64S(S) had more peaks distributed at CpG (O/E) ratios of 0 ~ 0.8, while the peaks of PA64S (F) were inclined to CpG (O/E) ratios of 1.0 ~ 1.8 (Figure 3b). The CpG O/E ratio is a widely used parameter to predict the DNA methylation level based on C → T transition mechanisms resulting from deamination of mCs over the course of evolution [2,28]. In our study, more peaks of PA64S (S) were distributed in 0 ~ 0.8 (Figure 3c), and the methylation level of PA64S (S) was significant higher than that of PA64S (F) (Figure 4). These results were consistent with previous studies, showing that genes with higher methylation levels tend to have lower CpG O/E ratios [28,29].

To get insight into the DNA methylation status of PA64S (S) and PA64S (F), RPKMs (Reads per 1 kb window per million mapped reads) were calculated for each sample and different elements (Figure 4). The results showed that PA64S(S) had higher methylation level than that of PA64S (F). And the DNA methylation levels between PA64S(S) and PA64S (F) were observed to be significantly different on the upstream 2k, 5’UTR, intron, 3’UTR, and downstream 2k regions of the gene body (*p* < 0.01, *p* < 0.001). However, there were not significant differences on CDS (coding DNA sequence) of the differential methylated regions (DMRs).

### DNA methylation patterns in genes and TE regions

Comparison of the gene methylation status showed that there were 725 genes hypermethylated and 533 genes hypomethylated in PA64S (S). Most of these DMRs were observed on the upstream 2k and downstream 2k of genes and many of them were hypermethylated in PA64S (S) (Table 2 and Figure 4). The number of hypermethylated genes showed significant difference between PA64S (S) and PA64S (F) in the upstream2k, 5’UTR, 3’UTR, and downstream 2k regions comparing with the hypomethylated genes (Table 2 and Figure 4). Interestingly, the differentially methylated genes of the CDS regions between PA64S (S) and PA64S (F) exhibited almost no differences (Table 2 and Figure 4).

**Table 2:** **Numbers of differentially methylated genes between PA64S(S) and PA64S (F) in different gene regions**

**Contrast**	**PA64S(S) VS. PA64S(F)- Hyper**	**PA64S(S) VS. PA64S(F)- Hypo**
Upstream 2k	245	122
5’UTR	25	10
CDS	86	84
Intron	93	69
3’UTR	23	10
Downstream 2k	253	106
Total	725	533

The significant enrichment of down-regulated genes in PA64S (S) was annotated (Table 3 and Additional file 2). These differential genes included: transposon, retrotransposon, phosphate carrier protein, NB-ARC domain containing protein, ent-kaurene synthase, PsbP and so on. They were involved in disease resistance, signal transduction, transport, chloroplast development and photosynthesis, energy metabolism, and pollen development. Further research on these genes may elucidate the functions of hypermethylation in the fertility transition of PA64S.

**Table 3:** **Significant GO enrichment of hypermethylated genes in PA64S (S)**

**Locus ID**	**DMR location**	**Annotation**	**p-value**
LOC_Os01g70080	Upstream 2k/ Intron	NB-ARC domain containing protein	1.43E-42
LOC_Os07g04950	Upstream 2k	Early nodulin 75-like protein	2.33E-29
LOC_Os01g70100	Upstream 2k/5'-UTR	zinc finger DHHC domain-containing protein	1.21E-22
LOC_Os09g38100	Upstream 2k	phosphate carrier protein, mitochondrial precursor	1.84E-15
LOC_Os09g39570	Upstream 2k	beta-amylase	2.29E-15
LOC_Os03g25840	Upstream 2k	Amino acid permease family protein	2.8E-14
LOC_Os12g30520	Upstream 2k	pumilio-family RNA binding repeat containing protein	1.06E-13
LOC_Os12g35730	Upstream 2k	transposon protein, putative, CACTA, En/Spm sub-class	1.36E-12
LOC_Os10g31780	Upstream 2k	oxidoreductase	2.25E-12
LOC_Os01g04330	Upstream 2k	Probable calcium-binding protein CML16	2.77E-11
LOC_Os06g42420	Upstream 2k	transposon protein, putative, unclassified	1.08E-10
LOC_Os01g70360	Upstream 2k	Long chain base biosynthesis protein 2b	1.21E-10
LOC_Os12g40130	Upstream 2k	phosphoribosyl transferase	6.86E-10
LOC_Os01g18620	Upstream 2k	transferase family protein	7.06E-10
LOC_Os12g30520	5'-UTR	pumilio-family RNA binding repeat containing protein	1.06E-13
LOC_Os04g30490	5'-UTR/ CDS/ Intron	MATE efflux family protein	3.05E-11
LOC_Os01g62890	5'-UTR	prenylated rab acceptor	3.75E-11
LOC_Os01g70540	CDS/ Intron	retrotransposon protein	3.94E-31
LOC_Os01g20880	CDS	OsWAK3	1.06E-15
LOC_Os01g70040	CDS/Intron	transposon protein, CACTA, En/Spm sub-class	1.08E-14
LOC_Os01g70030	CDS/Intron	transposon protein, CACTA, En/Spm sub-class	1.5E-14
LOC_Os04g02920	CDS/3'-UTR	leucine-rich repeat family protein	2.2E-14
LOC_Os04g35210	CDS	Leucine Rich Repeat family protein	9.11E-12
LOC_Os06g03850	CDS	impaired sucrose induction 1	8.63E-11
LOC_Os07g41610	CDS/Intron	retrotransposon protein, Ty1-copia subclass	1.86E-10
LOC_Os06g36540	CDS	retrotransposon protein, Ty3-gypsy subclass	9.81E-10
LOC_Os07g04940	Intron	PE-PGRS family protein precursor	2.33E-29
LOC_Os08g21920	Intron	retrotransposon protein	5.37E-23
LOC_Os06g21950	Intron	inorganic phosphate transporter	7.93E-15
LOC_Os12g19549	Intron	telomerase reverse transcriptase	1.36E-12
LOC_Os09g23650	Intron	FAM10 family protein	2.88E-11
LOC_Os06g03850	Intron	impaired sucrose induction 1	8.63E-11
LOC_Os10g30090	Intron	amino acid permease	4.79E-10
LOC_Os09g21770	3'-UTR	ES43 protein	1.03E-12
LOC_Os04g47410	3'-UTR	DHHC zinc finger domain containing protein	5.04E-12
LOC_Os01g70190	Downstream 2k	exostosin family domain containing protein	9.18E-65
LOC_Os01g70550	Downstream 2k	heparan-alpha-glucosaminide N-acetyltransferase	3.94E-31
LOC_Os12g36410	Downstream 2k	transposon protein, Pong sub-class	9.30E-24
LOC_Os01g70810	Downstream 2k	homeobox domain containing protein	2.97E-23
LOC_Os01g70270	Downstream 2k	auxin response factor	2.93E-21
LOC_Os04g12060	Downstream 2k	retrotransposon protein	2.75E-19
LOC_Os01g67980	Downstream 2k	cysteine proteinase EP-B 1 precursor	1.81E-16
LOC_Os01g71070	Downstream 2k	xylanase inhibitor	1.73E-15
LOC_Os09g39560	Downstream 2k	genetic modifier	2.29E-15
LOC_Os01g70020	Downstream 2k	DEK C terminal domain containing protein	3.62E-15
LOC_Os05g23610	Downstream 2k	protein phosphatase inhibitor 2 containing protein	2.20E-14
LOC_Os01g70690	Downstream 2k	Rapid ALkalinization Factor RALF family protein	7.42E-14
LOC_Os02g17780	Downstream 2k	ent-kaurene synthase, chloroplast precursor	8.65E-14
LOC_Os05g12210	Downstream 2k	chalcone synthase	1.15E-12
LOC_Os04g47420	Downstream 2k	transmembrane amino acid transporter protein	5.04E-12
LOC_Os07g17390	Downstream 2k	PsbP	2.81E-11
LOC_Os10g34910	Downstream 2k	secretory protein	3.37E-11
LOC_Os02g15690	Downstream 2k	polygalacturonase	5.54E-11
LOC_Os01g01840	Downstream 2k	helix-loop-helix DNA-binding domain containing protein	6.27E-11
LOC_Os06g40200	Downstream 2k	calcium-binding mitochondrial carrier	1.04E-10
LOC_Os01g70370	Downstream 2k	serine palmitoyltransferase 2	1.21E-10
LOC_Os01g45750	Downstream 2k	bile acid sodium symporter family protein	7.05E-10
LOC_Os01g18630	Downstream 2k	aspartic proteinase oryzasin-1 precursor	7.06E-10
LOC_Os06g36550	Downstream 2k	retrotransposon protein, Ty3-gypsy subclass	9.81E-10
LOC_Os01g70900	Downstream 2k	retrotransposon protein	9.84E-10

Transposable elements (TEs) are frequently methylated in plant genomes. The analysis of 26 repeat types revealed that LTR /Gypsy had the highest 5mC content in PA64S and was increased in PA64S (F) (Figure 3c). Further analysis of four types of LTR and LINE, SINE showed that all of the LTR retroelements were highly methylated in PA64S (F) (Table 4). In contrast, the two types of retroelements (LINE, SINE) had higher methylation in PA64S (S) than in PA64S (F) (Table 4).

**Table 4:** **Transposable element methylation of PA64S(S) and PA64S (F) based on 10 kb tiled genome windows**

**Type**	**PA64S(S)**		**PA64S(F)**	
	**reads**	**%**	**reads**	**%**
LTR/Copia	521,175	5.62	533,088	5.79
DNA/En-Spm	658,162	7.1	676,617	7.35
LTR/Gypsy	4,045,323	43.63	4,293,192	46.61
LTR	55,692	0.6	55,798	0.61
LINE/L1	174,518	1.88	171,516	1.86
SINE	167,785	1.81	154,945	1.68

### Validation of MedIP data by bisulfite sequencing

**Figure 5:**
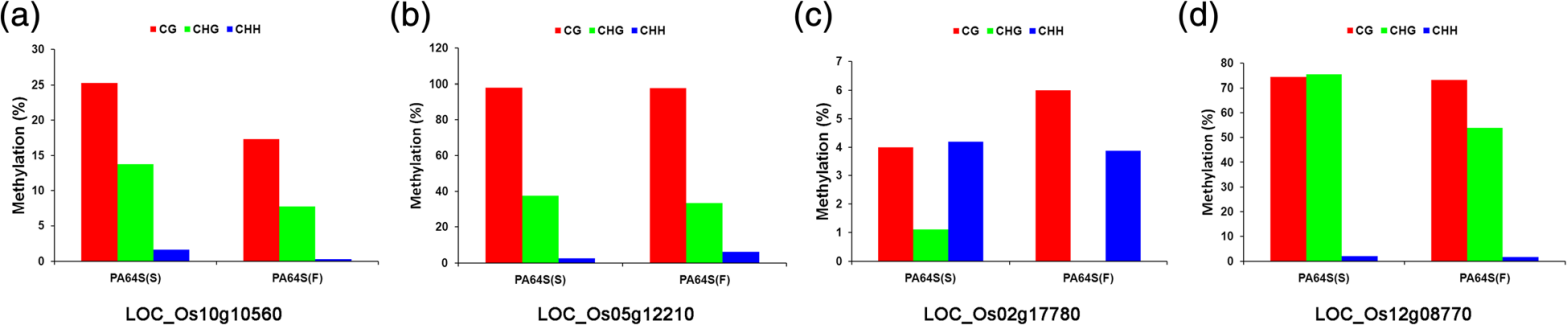
**DNA methylation patterns of four selected DMR-associated genes validated by bisulfite sequencing. (a)** LOC_Os10g10560 (invertase/pectin methylesterase inhibitor, IPMI); **(b)** LOC_Os05g12210 (chalcone synthase); **(c)** LOC_Os02g17780(ent-kaurene synthase, chloroplast precursor); and **(d)** LOC_Os12g08770 (photosystem I reaction center subunit N). The red, green and blue columns in the histograms refer to the collective methylation levels (in percentage) of CG, CHG, and CHH, respectively.

**Figure 6:**
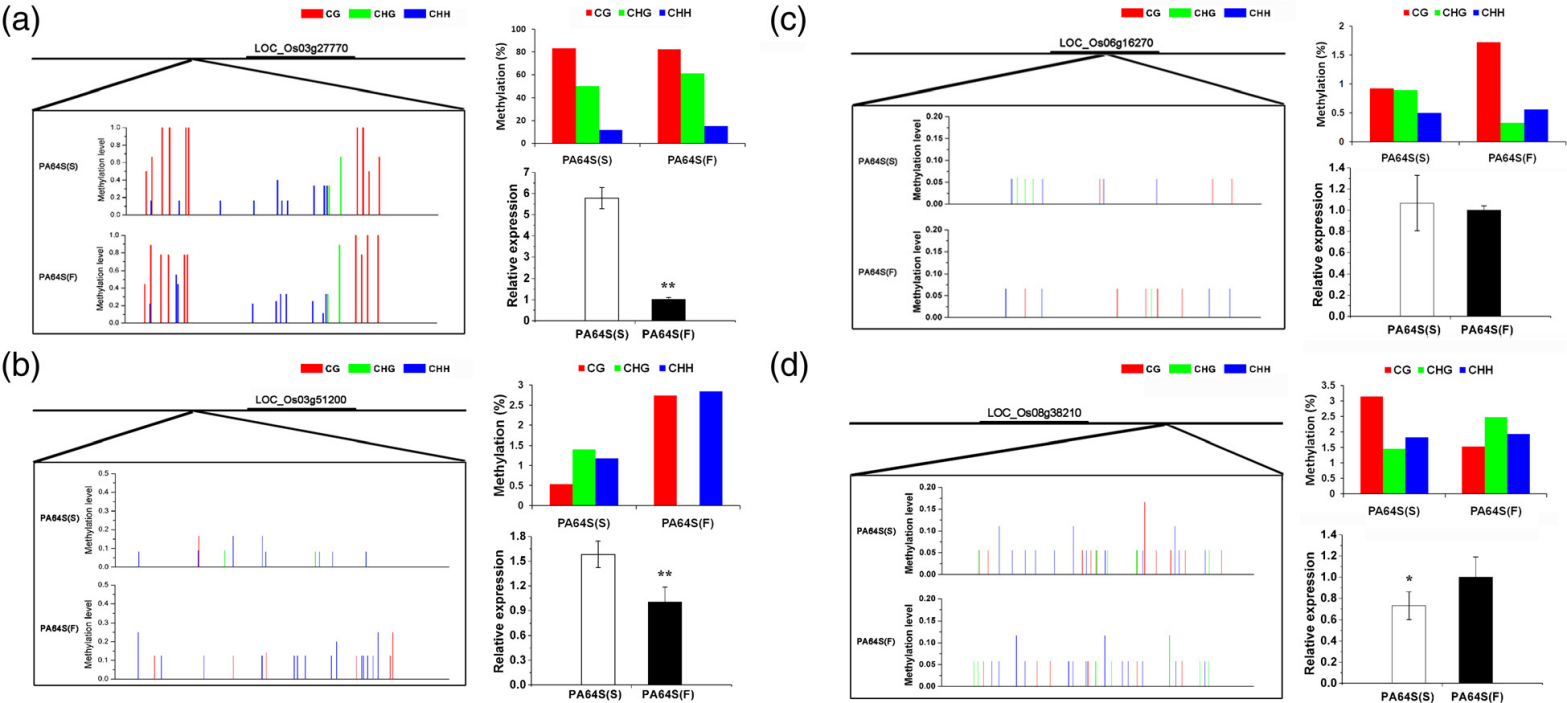
**DNA methylation patterns and gene expression of the four selected DMR-associated genes. (a)** Bisulfite sequencing and qRT-PCR analysis of LOC_Os03g27770 (heme oxygenase 2); **(b)** Bisulfite sequencing and qRT-PCR analysis of LOC_Os03g51200 (H2A); **(c)** Bisulfite sequencing and qRT-PCR analysis of LOC_Os06g16270 (heat shock factor binding protein 2); and **(d)** Bisulfite sequencing and qRT-PCR analysis of LOC_Os08g38210 (transcription factor BIM2). The red, green and blue columns in the histograms refer to the collective methylation levels (in percentage) of CG, CHG, and CHH, respectively.

In this study, the DNA methylation patterns of 20 DMR-associated genes were selected to carry out bisulfite sequencing for the validation of MedIP data, including LOC_Os10g10560 (invertase/pectin methylesterase inhibitor, IPMI), LOC_Os12g08770 (photosystem I reaction center subunit N), LOC_Os03g51200 (H2A), LOC_Os08g38210 (transcription factor BIM2) and so on (Figures 5 and 6). We found that many of the DMR-associated genes showed hyper-methylation in PA64S (S), and the results were almost in accordance with the MeDIP-seq data (Figures 5 and 6, and Additional file 3). For example, LOC_Os10g10560 (IPMI), LOC_Os03g01820 (vacuolar ATP synthase subunit E), LOC_Os06g23980 (MADS-AGL16) and LOC_Os04g37619 exhibited a higher methylation level in PA64S(S) compared with PA64S (F) (Additional file 3). However, the DMR-associated genes LOC_Os03g51200 (H2A) and LOC_Os03g27770 (heme oxygenase 2) were observed to be hypomethylation in PA64S(S) (Figure 6a, b).

### Association of DNA methylation and gene expression

Analysis of the relationship between DNA methylation and gene expression revealed that hypermethylation in some regions were related to the expression of neighboring genes. DNA methylation level of PA64S(S) on the upstream 2k, 5’UTR, intron, 3’UTR, and downstream 2k regions were hypermethylated compared with PA64S (F) (*p* < 0.001) (Figure 4b and Table 2). Correspondingly, many DMR-associated genes may be down-regulated in PA64S (S).

The DNA methylation patterns and gene expression of four DMR-associated genes, namely LOC_Os03g51200 (H2A), LOC_Os08g38210 (transcription factor BIM2), LOC_Os06g16270 (heat shock factor binding protein 2) and LOC_Os03g27770 (heme oxygenase 2), were further examined in this study (Figure 6). Three of these had lower DNA methylation levels, but higher expression in PA64S (S). Furthermore, The DMR-associated gene LOC_Os08g38210 showed a higher DNA methylation level and a lower expression in PA64S (S) (Figure 6d).

### Expression of the genes involved in brassinosteroid signaling pathway

**Figure 7:**
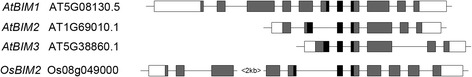
**The genomic organization of the rice** ***OsBIM2*** **(LOC_Os08g38210) loci is compared to those of the** ***BIM1*** **,** ***BIM2*** **and** ***BIM3*** **genes in Arabidopsis.** Note: This genomic organization is modified on the basis of Xing et al., 2013 [30].

**Figure 8:**
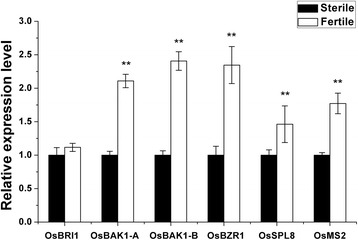
**Expression analysis of genes related to BR signaling pathway.**

One of the hypermethylated gene *OsBIM2* (LOC_Os08g38210), which is homolog with *BIM* gene family in Arabidopsis, was thought to be involved in BR signaling pathway. Comparison with the structure of *BIM1* suggested that *OsBIM2* had higher similarity to *BIM1* of Arabidopsis, with an extended *N*-terminal domain that was lacking in *BIM2* and *BIM3* [30]. Comparing the respective genomic loci also supported the idea that *OsBIM2* and Arabidopsis *BIM1* probably share a more ancestral intron-exon structure (Figure 7). And *BIM1* was reported to be cooperated with *BES1* to regulate BR-induced genes in Arabidopsis [26]. Therefore, we postulated that BR may participate in regulating male fertility in PA64S. To test this hypothesis, we examined the expression of some genes related to BR signaling pathway and the genes that regulated by BR. Expression analysis of these genes showed that some of them had lower expression in PA64S (S) (*P <* 0.01), including *OsBAK1*, *OsBZR1*, *OsSPL8* (Figure 8). The expression of *OsMS2*, one of the regulating genes for BR signaling in the tapetum and microspore development, was also dramatically reduced in PA64S (S) (*P <* 0.01) (Figure 8).

### Gene ontology (GO) and KEGG pathway enrichment analysis

In this study, genes that overlapped with the methylation peaks in the upstream 2k, downstream 2k or gene body regions were termed as methylated genes. A total of 36,321 methylated genes were found in the two samples: 32,678 in PA64S (S) and 32,990 in PA64S (F). Of them, 1258 methylated genes were identified to be differentially expressed genes between PA64S (S) and PA64S (F), and these included 725 genes that were hypermethylated and 533 genes that were hypomethylated in PA64S (S) (Table 2).

**Figure 9:**
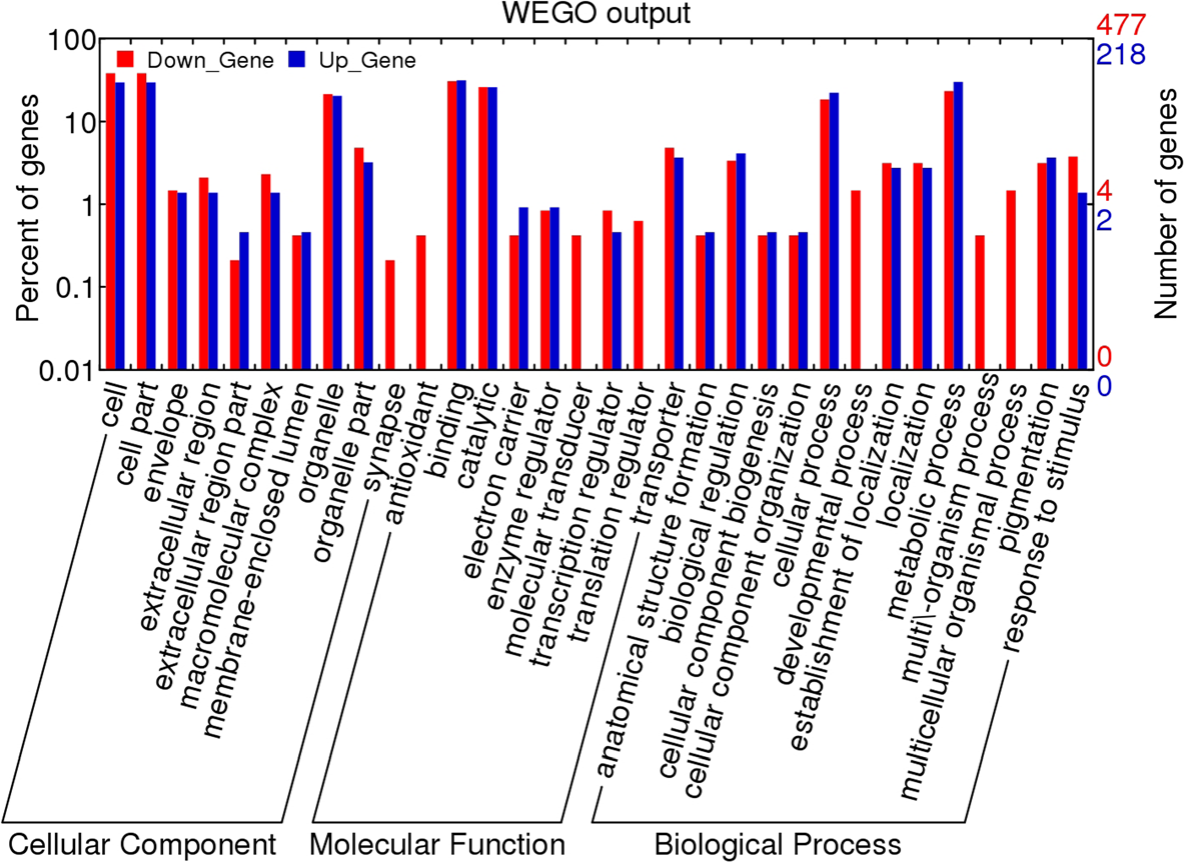
**Gene categories and distribution of differential methylated genes between PA64S(S) and PA64S (F).**

We further used WEGO (http://wego.genomics.org.cn) to functionally categorize the methylated genes and observed significant differences (Figure 9 and Additional file 4). Many genes enriched hypermethylation in PA64S(S), such as translation regulator, development process, multi-organism process and so on (Figure 9). The results showed that some genes were significantly down-regulated genes in PA64S (S), including 12 genes for “cellular component” (9), 16 genes for “molecular function” (21) and 64 genes for “biological process” (50) (Table 5 and Additional file 5). Methylated genes tend to be enriched in transferase activity, including transcription regulator as well as electron transporter. With respect to biological processes, the genes are enriched in functions associated with photorespiration, cellular metabolism, oxidative phosphorylation and mitochondrial ATP synthesis. Interestingly, the methylated genes of “response to stimulus” were increased in PA64S (S) (Figure 9). In contrast, for cellular component, the significantly differentially down-regulated genes in PA64S (S) were enriched in mitochondrial respiratory chain and mitochondrial inner membrane (Table 5).

**Table 5:** **Overrepresented Gene Ontology (GO) categories in the hypermethylated genes in PA64S (S)**

**GO term**	**Ontology** ^**a**^	**Element**	**Ontology description**	**Number in input list**	**Number in BG/Ref**	**p-value**
GO:0005750	C	Down2k_ Down	mitochondrial respiratory chain complex III	4	14	2.74e-05
GO:0005746	C	Down2k_ Down	mitochondrial respiratory chain	3	22	0.00104
GO:0005743	C	Down2k_ Down	mitochondrial inner membrane	5	78	0.00223
GO:0016742	F	3’-UTR_ Down	hydroxymethyl-, formyl- and related transferase activity	4	4	1.61e-14
GO:0016741	F	3’-UTR_ Down	transferase activity, transferring one-carbon groups	4	84	3.12e-08
GO:0030528	F	Intron_ Down	transcription regulator activity	4	178	1.09e-05
GO:0045153	F	Down2k_ Down	electron transporter, transferring electrons within CoQH2-cytochrome c reductase complex activity	4	13	4.58e-05
GO:0009853	P	3’-UTR_ Down	photorespiration	4	4	3.65e-13
GO:0046653	P	3’-UTR_ Down	tetrahydrofolate metabolic process	4	4	3.65e-13
GO:0006760	P	3’-UTR_ Down	folic acid and derivative metabolic process	4	8	2.56e-11
GO:0042558	P	3’-UTR_ Down	pteridine and derivative metabolic process	4	11	1.20e-10
GO:0043094	P	3’-UTR_ Down	cellular metabolic compound salvage	4	19	1.41e-09
GO:0006732	P	3’-UTR_ Down	coenzyme metabolic process	4	63	2.17e-07
GO:0009259	P	3’-UTR_ Down	ribonucleotide metabolic process	4	81	6.08e-07
GO:0051186	P	3’-UTR_ Down	cofactor metabolic process	4	102	1.55e-06
GO:0009117	P	3’-UTR_ Down	nucleotide metabolic process	4	111	2.19e-06
GO:0006725	P	3’-UTR_ Down	cellular aromatic compound metabolic process	4	129	4.02e-06
GO:0032787	P	3’-UTR_ Down	monocarboxylic acid metabolic process	4	142	5.94e-06
GO:0046483	P	3’-UTR_ Down	heterocycle metabolic process	4	189	1.88e-05
GO:0006122	P	Down2k_ Down	mitochondrial electron transport, ubiquinol to cytochrome c	4	6	2.35e-06
GO:0042775	P	Down2k_ Down	mitochondrial ATP synthesis coupled electron transport	4	18	0.00045
GO:0006119	P	Down2k_ Down	oxidative phosphorylation	4	28	0.00289
GO:0042773	P	Down2k_ Down	ATP synthesis coupled electron transport	4	28	0.00289

^a^, C: cellular component; F, molecular function; P, biological process.

To generate further insight view of pathway perturbation in PA64S (S) under long day length and high temperature conditions, we performed KEGG pathway analysis of the differentially methylated genes. As a result, 58 predicted pathways were enriched, including metabolism, circadian rhythm, photosynthesis, porphyrin and chlorophyll metabolism, peroxisome and oxidative phosphorylation (Additional files 6). Through the GO term enrichment and KEGG pathway analysis of the differentially regulated genes, we observed that certain genes were hypermethylated in PA64S (S) and that they were involved in photorespiration and mitochondrial ATP synthesis pathways. These pathways were closely connected to photosynthesis and energy synthesis. For instance, cytochrome c1-1 (LOC_Os05g23620) was involved in mitochondrial electron transport.

## Discussion

Although global DNA methylation surveys have been performed in some plants [3,31,32], little studies have been reported about the methylation profiles of PTGMS rice (for example PA64S) during the fertility transition. In this study, the objective was to perform a genome-wide identification of the methylated genes that affect the sterility-fertility transition in PTGMS rice PA64S. To confirm the results from MeDIP-seq, the methylation patterns of twenty regions in each sample were analyzed by bisulfite sequencing. And the methylation levels obtained by the two methods were generally in accord with each other. The read distribution analysis performed in this study found that uniquely mapped reads were enriched in the upstream 2k, 5’UTR, 3’UTR and downstream 2k regions. In addition, the rice sterile line PA64S(S) had higher methylation level than that of PA64S (F), and many DMR-associated genes were down-regulated in PA64S (S).

Transposable elements (TEs) are abundant features in plant genomes that are frequently marked by cytosine methylation. In this study, we examined many categories of transposable elements from the RepBase transposable element annotation database. LTR retroelements are the predominant type of transposable element sequences in plant genomes [33]. Our results showed that the LTR categories had higher 5mC content in PA64S (F) than in PA64S (S). And the LINE and SINE retroelement categories had an increased in 5mC content in PA64S (S) compared with PAS64 (F), although this difference was not significant. Previous studies using methylation-deficient mutants have demonstrated that hypomethylation can result in transposable element mobilization [34]. The increased 5mC content of TEs found in the present study suggested that the operation of adaptive genomic mechanisms might help protect genomes against transposable element spread. It would be of interest to measure the relationship of DNA methylation and TE elements during the fertility transition of PA64S.

In this study, the DNA methylation patterns of twenty DMR-associated genes were validated through bisulfite sequencing and we found that hypermethylation in some regions affected the expression of neighboring genes (Figures 5 and 6, Additional file 3). Although hypermethylated DMR-associated genes did not show significant enrichment of down-regulated genes, there were still some down-regulated genes with higher methylation levels (Figures 5 and 6). Some flower/anther development related genes were hypermethylated in PA64S (S), for example, LOC_Os06g23980 (putative MADS-box protein AGL16) and LOC_Os10g10560 (IPMI) (Additional file 3). Previous studies of a male sterile mutant (*Ms-cd1*) in *Brassica oleracea* have demonstrated that IPMI was specifically expressed in anther and filament and was not expressed in CMS NILs [35]. The increased 5mC content in IPMI of PA64S(S) found in the present study might lead to a lower expression of IPMT and most likely disturbed the anther cell wall metabolism. Furthermore, photosystem and mitochondrial ATP synthesis associated genes, including LOC_Os12g08770 (photosystem I reaction center subunit N), LOC_Os07g17390 (PsbP) and LOC_Os03g01820 (vacuolar ATP synthase subunit E) were also hypermethyalted in PA64S (S) (Additional file 2). High temperature (>23.5°C) and LD (14 h) conditions increased the methylation levels of these genes and down-regulated their expression during anther development in PA64S, which might in turn lead to PA64S sterility.

In contrast, some heat shock proteins, such as LOC_Os08g43490 (heat shock protein DnaJ), LOC_Os06g16270 (heat shock factor binding protein 2) were found to be hypomethylated in PA64S (S) in our study, and the expression level of LOC_Os06g16270 was also higher in PA64S (S) (Figure 6c). Because heat shock proteins are always expressed in response to stress, their higher expression in PA64S (S) suggested that the adverse environment might affect anther development. These findings suggested that different environments might regulate the gene expression through DNA methylation in the gene body or flanking sequence of fertility associated genes.

In Arabidopsis and rice, the BR signaling pathway is similar. Generally, BRs bind the extracellular domain of BRI1, and then BRI1 heteromerizes with the BRI1-associated kinase 1 (BAK1). The receptor complex then activates the BR-signaling kinases (BSKs) and inactivates the glycogen synthase kinase-3-like kinase BIN2, resulting in nuclear accumulation of unphosphorylated forms of two transcription factors BZR1 and BES1, which activate the expression of BR-responsive genes [36-38]. In this study, some of the components in BR signaling pathway, such as *OsBRI*, *OsBAK1*, *OsBZR* and *OsBIM2* were dramatically reduced in male sterile line PA64S (S) (Figure 8). Our results indicated that BR signaling involved in regulating the sterility-fertility transition of PA64S under different environments.

On the other hand, *OsBIM2* (LOC_Os08g38210) showed a higher DNA methylation level in PA64S(S) and its expression dramatically reduced (Figure 6d). In Arabidopsis, *BIM1* and *SPL8* act cooperatively in a common complex or via synergistic pathways to promote male fertility [30]. In our study, both *OsBIM2* and Os*SPL8* were downregulated in PA64S(S) (Figure 8). Since *OsBIM2* is homolog with *BIM1*, the lower expression level will affect BR signaling pathway and then regulated the downstream genes which involved in anther and pollen development. Therefore, DNA methylation of BR signaling related genes may affect the expression of key genes (for example, *OsMS2*) for anther development, leading to male sterility under long-day and high temperature conditions. However, whether the hypermethylated *OsBIM2* directly suppress key genes need to be further investigated.

The GO term significant enrichment analysis showed that many genes, including the transposons and retrotransposons, were hypermethylated in PA64S (S) (Tables 3 and 5 and Additional files 3 and 4). For example, LOC_Os09g38100 (phosphate carrier protein mitochondrial precursor) and LOC_Os06g40200 (calcium-binding mitochondrial carrier) were observed higher methylation levels in PA64S (S) than PA64S (F) (Table 3 and Additional file 3). These two genes participate in mitochondrial respiratory chain and facilitate mitochondrial ATP synthesis [39]. Thus, their hypermethylated would affect the energy synthesis during pollen development. Another hypermethylated gene LOC_Os02g17780 (ent-kaurene synthase, chloroplast precursor) was an early intermediate in GA biosynthesis [40]. Other significantly differentially expressed DMR-associated genes included LOC_Os12g40130 (phosphoribosyl transferase), LOC_Os04g30490 (MATE efflux family protein) and LOC_Os05g12210 (chalcone synthase). Some studies have reported that the adenine phosphoribosyl transferase activity deficient mutants of *Arabidopsis thaliana* were male sterile due to abortion of pollen development after the meiotic divisions of pollen mother cells [41]. The MATE (Multidrug And Toxic Compound Extrusion) efflux protein family has also been reported to be differentially expressed in sterile and fertile NILs of *Brassica oleracea* [35]. Chalcone synthase catalyzes the initial step of the branch of phenylpropanoid pathway which leads to flavonoids. The absence of chalcone synthase activity was found to have a pleiotropic effect in maize and petunia mutants, and pollen fertility/pollen germination and flavonoid synthesis was disrupted [42]. Therefore, these differentially expressed genes might be involved in fertility transition.

To uncover the regulatory mechanisms of the fertility transition, the regulatory network of differentially methylated genes was examined in this study. Several important pathways were found, including flavone and flavonol biosynthesis, circadian rhythm, photosynthesis, porphyrin and chlorophyll metabolism, and oxidative phosphorylation (Additional files 6). Previous studies have reported that flavone and flavonol biosyntheses are associated with pollen development. Flavonoids are required for pollen viability in many species [42-44]. In addition, flavonols can promote pollen germination frequency and pollen tube growth in tobacco [45]. Many higher plants use the day length as an environmental cue to switch from vegetative to reproductive growth and plants with defects in their circadian regulation cannot properly regulate the timing of the floral transition [46]. In PGMS rice, the circadian rhythm genes *OsPRR37* in inflorescences have been reported to directly affect the male sterility transition in Nongken 58S [22]. In this study, some DMR-associated genes, such as LOC_Os05g12210 (chalcone synthase) and LOC_Os08g38210 (transcription factor *BIM2*), were found to be involved in the circadian rhythm. The other three pathways, namely photosynthesis, porphyrin and chlorophyll metabolism, and oxidative phosphorylation were related to carbon or energy metabolism [47,48]. The down-regulated genes of these pathways in PA64S (S) may lead to insufficient energy and materials for pollen development. Therefore, these five pathways were regarded as potentially related to the fertility transition of PA64S in the present study.

## Conclusions

We systematically investigated the complete DNA methylome of photoperiod- and thermo-sensitive male sterile rice Peiai 64S under two different environments (different temperatures and day lengths). From the whole-genome DNA metylation map, hypermethylation was observed in PA64S (S) and many pathways such as circadian rhythm, photosynthesis and oxidative phosphorylation were participated in the fertility transition of PA64S. Furthermore, the hypermethylated *OsBIM2* may be involved in brassinosteroid signaling pathway and affected the male fertility in PA64S.

## Methods

### Plant materials

The rice photo-thermo-sensitive genic male sterile line PA64S, which was maintained at Wuhan University, was used in this study. The seeds of PA64S were sown under two different natural ecological conditions: in Hainan (18°48′ N, 110°02′ E) from December, 2010 to March, 2011 (fertility, F), and in Wuhan (30°30′ N, 114°18′ E) from May 2011 to August 2011 (sterility, S). Young panicles of PA64S of the two phenotypes at the meiosis stage were collected, and the pollen fertility was assayed by staining with potassium iodide (1% I_2_-KI).

### DNA preparation and methylated DNA immunoprecipitation

Total genomic DNA was extracted using a Genomic DNA Miniprep Kit (Axygen) following the manufacturer’s instructions. The DNA quality was then evaluated by agarose gel electrophoresis and a BioPhotometer Plus spectrophotometer (Eppendorf, Germany).

Subsequently, the two samples were sonicated to produce DNA fragments ranging from 100 to 500 bp. After end repairing, phosphorylating and A-tailing with Paired-End DNA Sample Prep kit (Illumina, USA), the DNA was ligated to an Illumina sequencing primer adaptor. Then the fragments were used for methylated DNA immunoprecipitation (MeDIP) enrichment using a Magnetic Methylated DNA Immunoprecipitation kit (Diagenod, Belgium) following the manufacturer’s recommendations and the qualifying DNA was used for PCR amplification. Then bands between 220 and 320 bp were excised from the gel and purified with a QIAquick Gel Extraction Kit (Qiagen, Germany). The products were quantified with a Quant-iT™ dsDNA HS Assay Kit (Invitrogen, USA) using an Agilent 2100 Analyzer (Agilent Technologies, USA). Following qPCR qualification, DNA libraries were sequenced on the Illumina HiSeq 2000 (Illumina, CA, USA) to generate paired-end 50-bp reads by the Beijing Genomics Institute (BGI, China).

### Bioinformatic analysis

The raw data obtained from Illumina sequencing were first processed to filter out reads containing adapters, unknown or low quality bases and were then were mapped to the rice reference genome (ftp://ftp.plantbiology.msu.edu/pub/data/Eukaryotic_Projects/o_sativa) by SOAPaligner v 2.21 (http://soap.genomics.org.cn/) with no more than 2 bp mismatches [49]. The uniquely mapped data were retained for read distribution analysis, including the distribution in rice chromosomes and the distribution in different components of the genome.

Subsequently, the regions of differential methylation (DMRs) with DNA methylation peaks were employed for differential DNA methylation analysis. Methylated regions were deemed significant differentially methylated between PA64S (S) and PA64S (F) with a *p*-value < 0.05, a false discovery rate (FDR) < 0.05 and at least a 2.0-fold change in sequence counts. The base data manipulations and statistical analysis were performed using the R package (http://www.r-project.org). Significant differences of DNA methylation level between PA64S (S) and PA64S (F) were evaluated using Student’s *t* test.

### Gene Ontology and KEGG pathway analysis

Significantly DMRs in the whole gene body (DMGs) common to comparisons between PA64S (S) and PA64S (F) with opposing directions of methylation difference (for example, down-methylated or up-methylated in PA64S (S) relative to PA64S (F)) were screened and annotated with Gene Ontology (GO), and functionally annotated through KEGG Orthology (KO) enrichment analysis. The GO enrichment analysis of the functional significance applies a hypergeometric test to map all of the differentially expressed genes (DEGs) to terms in the GO database, looking for significantly enriched GO terms in the differentially expressed genes compared with the complete genome. The formula used in this analysis is the following:

$$ \mathrm{P}=1\hbox{-} {\displaystyle \sum_{i=0}^{m-1}\frac{\left({}_i^M\right)\left({}_{n-i}^{N-M}\right)}{\left({}_n^N\right)}} $$

where *N* is the number of all genes with a GO annotation in the rice genome, *n* is the number of those N genes that are differentially expressed, *M* is the number of all of the genes that are annotated to certain GO terms, and *m* is the number of differentially expressed genes in *M*. The hypergeometric test was also performed using a user written program in R (R Core Team, Vienna, Austria). The KEGG pathway enrichment analysis identifies significantly enriched metabolic pathways or signal transduction pathways in differentially expressed genes comparing with the whole genome background. The calculating formula and the applied program were the same as that used in the GO analysis. Then, FDR (Q-value) ≤0.05 was used as the threshold to determine the most significantly enriched pathways in DEGs.

### Bisulfite sequencing

Genomic DNA was modified using an EpiTect Bisulfite kit (Qiagen, Germany) according to the manufacturer’s instructions. Briefly, 1 μg of DNA was first treated with bisulfite containing a C-T conversion reagent and then incubated at 95°C for 5 min, and 64°C for 5 h. The modified DNA was purified using a MinElute DNA spin columns (Qiagen, Germany) and stored at -20°C until use. Primers for selected genic sequences for bisulfite sequencing were designed by the Meth-Primer Program (http://www.urogene.org/methprimer/) and were given in Additional file 7. For each PCR reaction, 1.0 μl of bisulfite treated DNA was used in a 50 μl reaction system. These PCR products were gel-purified using a Gel Extraction Kit (Omega, USA), and then cloned into the pMD18-T vector (Takara, Dalian, China) and sequenced. At least fifteen clones were sequenced for each sample. The methylation levels which are expressed as percentage (%) per site for each of the three types of cytosines, CG, CHG and CHH, were calculated by dividing the number of non-converted (methylated) cytosines by the total number of cytosines within the assay. The analyses of the bisulfite sequencing results were conducted using the Kismeth website (http://katahdin.mssm.edu/kismeth).

### RNA isolation and real time RT-PCR

Total RNA was extracted from young panicles using an RNA isolation kit (BioDev, Beijing, China) and treated with DNase I (Fermentas, Canada) to remove the genomic DNA contamination. Approximately 2 μg of RNA from each sample was used for reverse transcription using the RevertAid First Strand cDNA Synthesis Kit (Fermentas, Canada). The reaction was carried out in a total volume of 20 μl, including 2 μg of RNA, 1 μl of oligo (dN)18 primers (100 μmol/L), 4 μl 5× reaction buffer, 2 μl 10 mmol/L dNTP mix, 1 μl of RiboLock RNase Inhibitor (20 U/μl), and 1 μl of M-MuLV Reverse Transcriptase (Fermentas, Canada). The procedure was as follows: the mixture was first denatured at 70°C for 10 min and then incubated at 37°C for 5 min, followed by 42 °C for 60 min and at last incubated at 70°C for 15 min.

Quantitative real-time PCR (qPCR) was performed using a StepOne Plus real-time PCR system (Applied Biosystems) in a total volume of 10 μl reaction mixture, which included 1 μl cDNA, 0.4 μl each primer (5 μmol/L each), 0.2 μL ROX Reference Dye (50×) and SYBR premix Ex Taq (Tli RNaseH plus) (2×) (Takara, Dalian, China). The primers used for the qPCR experiments are given in Additional file 7. The qPCR reactions were repeated three times for each sample from three independent experiments. After the amplification steps, the melting curve was determined for each primer pair to verify that only one specific product had been amplified. Relative quantification was performed using the 2^△△^Ct method [50]. Rice *actin1* was used as a reference for the mRNA expression analyses.

### Availability of supporting data

All MeDIP-seq raw data supporting the results of this article were deposited in the sequence read archive repository (http://www.ncbi.nlm.nih.gov/sra/) under Sequence Read Archive (SRA) database (accession # SRP050401).

## Additional files

Additional file 1:
**Chromosome distribution of reads in PA64S (S) and PA64S (F).** The distribution of reads in the chromosomes 1-12 of the rice genome was shown with a red color for each sample. The MeDIP-seq reads were plotted in 10 kb windows along chromosome. Additional file 2:
**Annotation of DMR-associated genes between PA64S (S) and PA64S (F).**
Additional file 3:
**Bisulfite sequencing of twenty selected DMR-associated genes.** The red, green and blue columns in the histograms refer to the collective methylation levels (in percentage) of CG, CHG, and CHH, respectively. Additional file 4:
**Overrepresented Gene Ontology (GO) categories in genes that are hypermethylated in PA64S(S).**
Additional file 5:
**KEGG pathways of all of the differentially methylated genes between PA64S(S) and PA64S (F).**
Additional file 6:
**Pathways related to the sterility-fertility transition in PA64S.**
Additional file 7:
**Primers for bisulfite sequencing and qRT-PCR.**

## References

[1] Feng S, Jacobsen SE, Reik W (2010). Epigenetic reprogramming in plant and animal development. Science.

[2] Xiang H, Zhu J, Chen Q, Dai FY, Li X, Li M, Zhang HY, Zhang GJ, Li D, Dong Y, Zhao L, Lin Y, Cheng DJ, Yu J, Sun JF, Zhou XY, Ma KL, He YH, Zhao YX, Guo SC, Ye MZ, Guo GW, Li YR, Li RQ, Zhang XQ, Ma LJ, Kristiansen K, Guo QH, Jiang JH, Beck S, Xia QY, Wang W, Wang J (2010). Single base-resolution methylome of the silkworm reveals a sparse epigenomic map. Nat Biotechnol.

[3] Zhang X, Yazaki J, Sundaresan A, Cokus S, Chan SWL, Chen HM, Henderson IR, Shinn P, Pellegrini M, Jacobsen SE, Ecker JR (2006). Genome wide High-Resolution Mapping and Functional Analysis of DNA Methylation in Arabidopsis. Cell.

[4] Ruike Y, Imanaka Y, Sato F, Shimizu K, Tsujimoto G (2010). Genome-wide analysis of aberrant methylation in human breast cancer cells using methyl-DNA immune- precipitation combined with high-throughput sequencing. BMC genomics.

[5] Cokus SJ, Feng S, Zhang X, Chen Z, Merriman B, Haudenschild CD, Pradhan S, Nelson SF, Pellegrini M, Jacobsen SE (2008). Shotgun bisulphitesequencing of the Arabidopsis genome reveals DNA methylation patterning. Nature.

[6] Lister R, O’Malley RC, Tonti-Filippini J, Gregory BD, Berry CC, Millar AH, Ecker JR (2008). Highly integrated single-base resolution maps of the epigenome in Arabidopsis. Cell.

[7] Zhang CJ, Chu HJ, Chen GX, Shi DW, Zuo M, Wang J, Lu CG, Wang P, Chen L (2007). Photosynthetic and biochemical activities in flag leaves of a newly developed super high-yield hybrid rice (*Oryza sativa*) and its parents during the reproductive stage. J Plant Res.

[8] Reinders J, Wulff BB, Mirouze M, Mari-Ordonez A, Dapp M, Rozhon W, Bucher E, Theiler G, Paszkowski J (2009). Compromised stability of DNA methylation and transposon immobilization inmosaic Arabidopsis epigenomes. Genes Dev.

[9] Cheng C, Daigen M, Hirochika H (2006). Epigenetic regulation of the rice retrotransposon *Tos17*. Mol Genet Genomics.

[10] Kashkush K, Khasdan V (2007). Large-scale survey of cytosine methylation of retrotransposons and the impact of readout transcription from long terminal repeats on expression of adjacent rice genes. Genetics.

[11] Down TA, Rakyan VK, Turner DJ, Flicek P, Li H, Kulesha E, Gräf S, Johnson N, Herrero J, Tomazou EM, Thorne NP, Bäckdahl L, Herberth M, Howe KL, Jackson DK, Miretti MM, Marioni JC, Birney E, Hubbard TJP, Durbin R, Tavaré S, Beck S (2008). A Bayesian deconvolution strategy for immunoprecipitation-based DNA methylome analysis. Nat Biotechnol.

[12] Tomazou EM, Rakyan VK, Lefebvre G, Andrews R, Ellis P, Jackson DK, Langford L, Francis MD, Bäckdah L, Miretti M, Coggill P, Ottaviani D, Sheer D, Murrell A, Beck S (2008). Generation of a genomic tiling array of the human major histocompatibility complex (MHC) and its application for DNA methylation analysis. BMC Med Genomics.

[13] Delseny M, Salses J, Cooke R, Sallaud C, Regad F, Lagoda P, Guiderdoni E, Ventelon M, Brugidou C, Ghesquiere A (2001). Rice genomics: Present and future. Plant Physiol Bioch.

[14] Yuan LP (1994). Purification and production of foundation seed of rice PGMS and TGMS lines. Hybrid Rice.

[15] Xu JJ, Wang BH, Wu YH, Du PN, Wang J, Wang M, Yi CD, Gu MH, Liang GH (2011). Fine mapping and candidate gene analysis of *ptgms2-1*, the photoperiod- thermo- sensitive genic male sterile gene in rice (*Oryza sativa* L.). Theor Appl Genet.

[16] Yuan LP (1990). Progress of two-line system hybrid rice breeding. Sci Agric Sin.

[17] Yang Q, Liang C, Zhuang W, Li J, Deng HB, Deng QY, Wang B (2007). Characterization and identification of the candidate gene of rice thermo-sensitive genic male sterile gene *tms5* by mapping. Planta.

[18] Shi MS (1985). The discovery and study of the photosensitive recessive male sterile rice. Sci Agric Sin.

[19] Wang WW, Meng B, Ge XM, Song SH, Yang Y, Yu XM, Wang LG, Hu SN, Liu SQ, Yu J (2008). Proteomic profiling of rice embryos from a hybrid rice cultivar and its parental lines. Proteomics.

[20] Xu ML, Zhou GQ, Chen LB (1999). Response of fertility of Pei’ai 64S to temperature and photoperiod in rice. Sci Agric Sin.

[21] Zhou H, Liu QX, Li J, Jiang DG, Zhou LY, Wu P, Lu S, Li F, Zhu LY, Liu ZL, Chen LT, Liu YG, Zhuang CX (2012). Photoperiod and thermo-sensitive genic male sterility in rice are caused by a point mutation in a novel noncoding RNA that produces a small RNA. Cell Res.

[22] Wang W, Liu ZW, Guo ZB, Song GY, Cheng Q, Jiang DM, Zhu YG, Yang DC (2011). Comparative transcriptomes profiling of photoperiod sensitive male sterile rice Nongken 58S during the male sterility transition between short-day and long-day. BMC Genomics.

[23] Ding JH, Shen JQ, Mao HL, Xie WB, Li XH, Zhang QF (2012). RNA-directed DNA methylation is involved in regulating photoperiod sensitive male sterility in rice. Mol Plant.

[24] Kutschera U, Wang ZY (2012). Brassinosteroid action in flowering plants: A darwinian perspective. J Exp Bot.

[25] Ye QQ, Zhu WJ, Li L, Zhang SS, Yin YH, Ma H, Wang XL (2010). Brassinosteroids control male fertility by regulating the expression of key genes involved in *Arabidopsis* anther and pollen development. Proc Natl Acad Sci USA.

[26] Yin YH, Vafeados D, Tao Y, Yoshida SG, Asami T, Chory J (2005). A new class of transcription factors mediates brassinosteroid-regulated gene expression in *Arabidopsis*. Cell.

[27] Nemhauser JL, Mockler TC, Chory J (2004). Interdependency of brassinosteroid and auxin signaling in Arabidopsis. PLoS Biol.

[28] Elango N, Hunt BG, Goodisman MA, Yi SV (2009). DNA methylation is widespread and associated with differential gene expression in castes of the honeybee, *Apis mellifera*. Proc Natl Acad Sci USA.

[29] Suzuki MM, Kerr AR, De Sousa D, Bird A (2007). CpG methylation is targeted to transcription units in an invertebrate genome. Genome Res.

[30] Xing SP, Quodt V, Chandler J, Hohmann S, Berndtgen R, Huijser P (2013). SPL8 acts together with the brassinosteroid-signaling component BIM1 in controlling Arabidopsis thaliana male fertility. Plants.

[31] Li X, Zhu JD, Hu FY, Ge S, Ye MZ, Xiang H, Zhang GJ, Zheng XM, Zhang HY, Zhang SL, Li Q, Luo RB, Yu C, Yu J, Sun JF, Zou XY, Cao XF, Xie XF, Wang J, Wang W (2012). Single-base resolution maps of cultivated and wild rice methylomes and regulatory roles of DNA methylation in plant gene expression. BMC Genomics.

[32] Vining K, Pomraning KR, Wilhelm LJ, Priest HD, Pellegrini M, Mockler TC, Freitag M, Strauss SH (2012). Dynamic DNA cytosine methylation in the *Populus trichocarpa* genome: tissue-level variation and relationship to gene expression. BMC Genomics.

[33] Kumar A, Bennetzen JL (1999). Plant retrotransposons. Annu Rev Genet.

[34] Vining K, Pomraning KR, Wilhelm LJ, Ma C, Pellegrini M, Di YM, Mockler TC, Freitag M, Strauss SH (2013). Methylome reorganization during in vitro dedifferentiation an regeneration of *Populus trichocarpa*. BMC Plant Biol.

[35] Lou P, Kang J, Zhang G, Bonnema G, Fang Z, Wang X (2007). Transcript profiling of a dominant male sterile mutant (*Ms-cd1*) in cabbage during flower bud development. Plant Sci.

[36] Clouse SD (2011). Brassinosteroid signal transduction: from receptor kinase activation to transcriptional networks regulating plant development. Plant Cell.

[37] He JX, Gendron JM, Yang YL, Li JM, Wang ZY (2002). The GSK3-like kinase BIN2 phosphorylates and destabilizes BZR1, a positive regulator of the brassinosteroid signaling pathway in Arabidopsis. Proc Natl Acad Sci USA.

[38] Wang XL, Chory J (2006). Brassinosteroids regulate dissociation of BKI1, a negative regulator of BRI1 signaling, from the plasma membrane. Science.

[39] Stael S, Rocha AG, Robinson AJ, Kmiecik P, Vothknecht UC, Teige M (2011). Arabidopsis calcium-binding mitochondrial carrier proteins as potential facilitators of mitochondrial ATP-import and plastid SAM-import. FEBS Lett.

[40] Yamaguchi S, Sun TP, Kawaide H, Kamiya Y (1998). The GA2 Locus of Arabidopsis thaliana Encodes ent-Kaurene Synthase of Gibberellin Biosynthesis. Plant physiol.

[41] Moffatt B, Somerville C (1988). Positive selection for male-sterile mutants of Arabidopsis lacking adenine phosphoribosyl transferase activity. Plant physiol.

[42] Mo YY, Nagel C, Taylor LP (1992). Biochemical complementation of chalcone synthase mutants defines a role for flavonols in functional pollen. Proc Natl Acad Sci USA.

[43] Taylor LP, Jorgensen R (1992). Conditional male-fertility in chalconesynthase -deficient petunia. J Hered.

[44] Buer CS, Imin N, Djordjevic MA (2010). Flavonoids: new roles for old molecules. J Integr Plant Biol.

[45] Ylstra B, Touraev A, Moreno RMB, Stöger E, van Tunen AJ, Vicente O, Joseph NMM, Heberle-Bors E (1992). Flavonols stimulate development, germination, and tube growth of tobacco pollen. Plant physiol.

[46] Doyle MR, Davis SJ, Bastow RM, McWatters HG, Kozma-Bognár L, Nagy F, Millar AJ, Amasino RM (2002). The ELF4 gene controls circadian rhythms and flowering time in *Arabidopsis thaliana*. Nature.

[47] Bazzaz FA, Carlson RW, Harper JL (1979). Contribution to reproductive effort by photosynthesis of flowers and fruits. Nature.

[48] Hatefi Y (1985). The mitochondrial electron transport and oxidative phosphorylation system. Annu Rev Biochem.

[49] Li R, Li Y, Kristiansen K, Wang J (2008). SOAP: short oligonucleotide alignment program. Bioinformatics.

[50] Livak KJ, Schmittgen TD (2001). Analysis of relative gene expression data using real-time quantitative PCR and the 2(T) (-Delta Delta C) method. Methods.

