# Supplementary material for: Genome-wide analysis of DNA methylation in photoperiod- and thermo-sensitive male sterile rice Peiai 64S
Source: BMC Genomics. 2015 Feb 19;16(1):102. doi: 10.1186/s12864-015-1317-7 (PMC4367915; doi:10.1186/s12864-015-1317-7)
Supplement: Additional file 1: — Chromosome distribution of reads in PA64S (S) and PA64S (F). The distribution of reads in the chromosomes 1-12 of the rice genome was shown with a red color for each sample. The MeDIP-seq reads were plotted in 10 kb windows along chromosome. [file 12864_2015_1317_MOESM1_ESM.pdf]

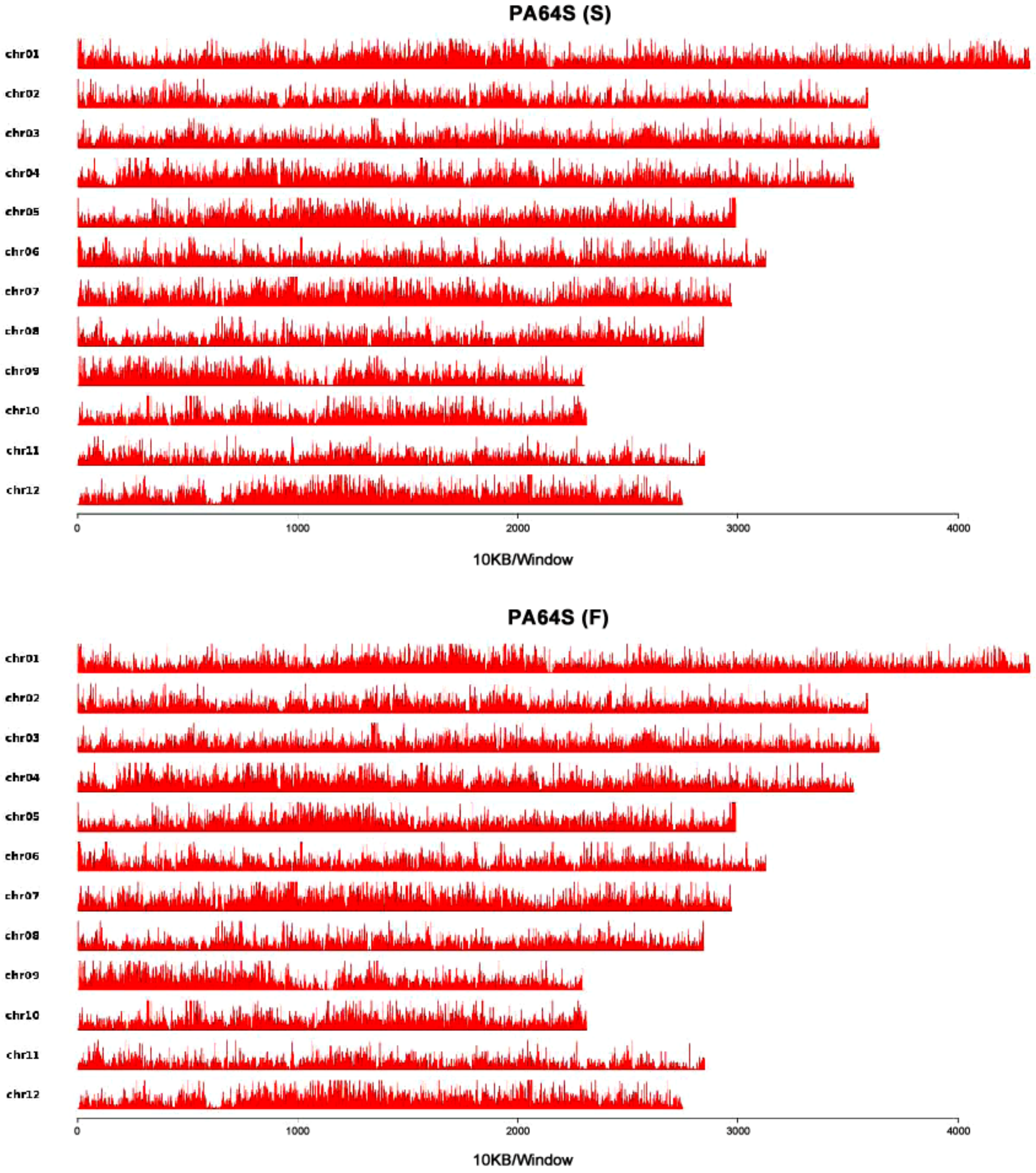

Additional file 1. Chromosome distribution of reads in PA64S (S) and PA64S (F). The distribution of reads in the chromosomes 1-12 of the rice genome was shown with a red color for each sample. The MeDIP-seq reads were plotted in 10 kb windows along chromosome.
