# Supplementary material for: Genome-wide analysis of DNA methylation in photoperiod- and thermo-sensitive male sterile rice Peiai 64S
Source: BMC Genomics. 2015 Feb 19;16(1):102. doi: 10.1186/s12864-015-1317-7 (PMC4367915; doi:10.1186/s12864-015-1317-7)

Additional file 3 Bisulfite sequencing for twenty selected DMR-associated genes.

LOC_Os03g01820: vacuolar ATP synthase subunit E (AT) **S: PA64S(S) F: PA64S(F)**

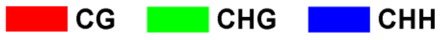

AT-S

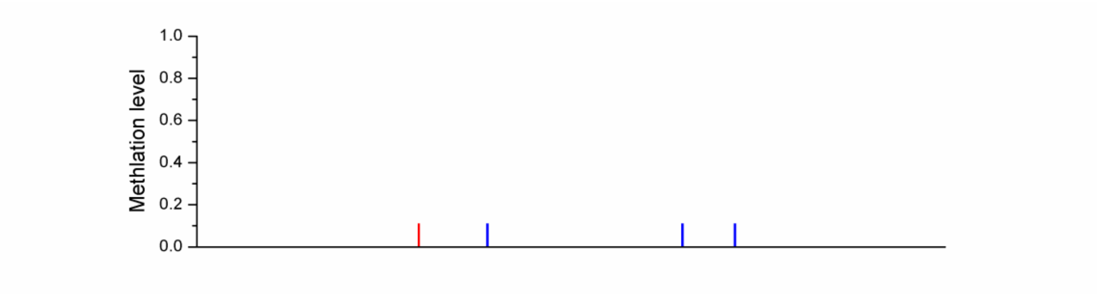

AT-F

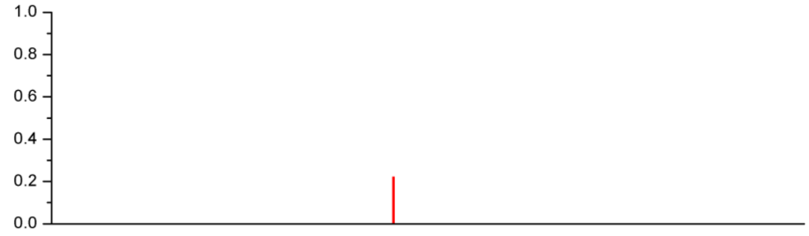

LOC_Os08g43490: heat shock protein DnaJ, putative, expressed (DJ)

DJ-S

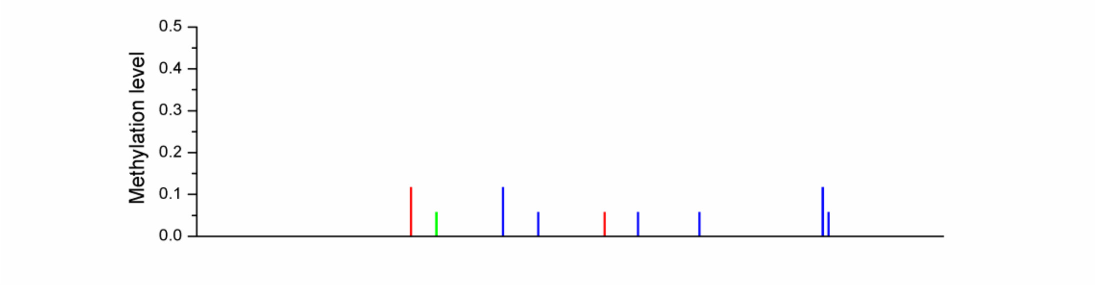

DJ-F

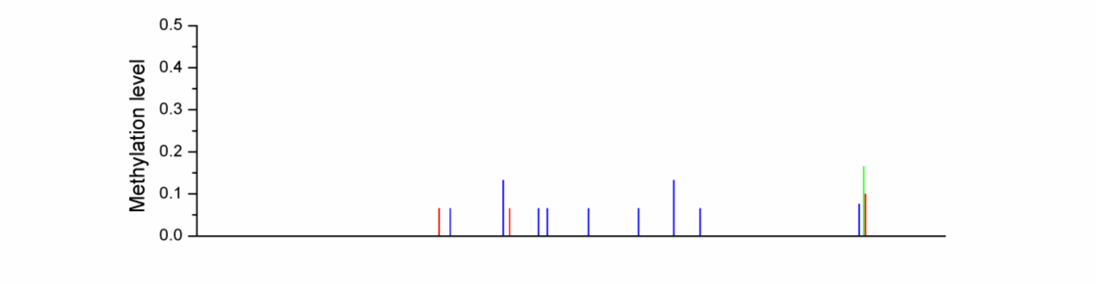

LOC_Os03g51200: H2A

H2A-S

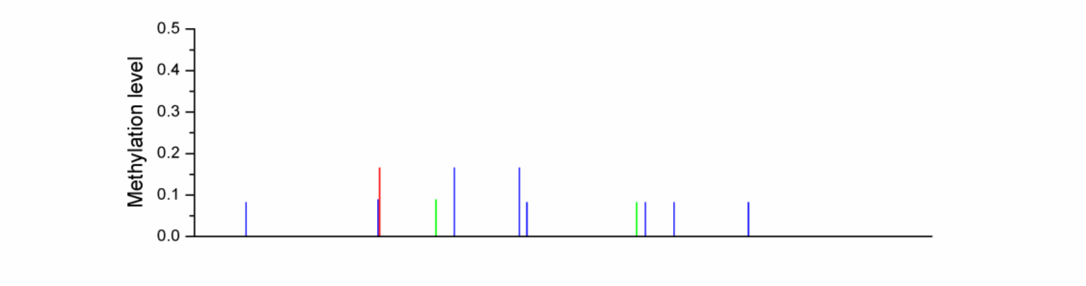

H2A-F

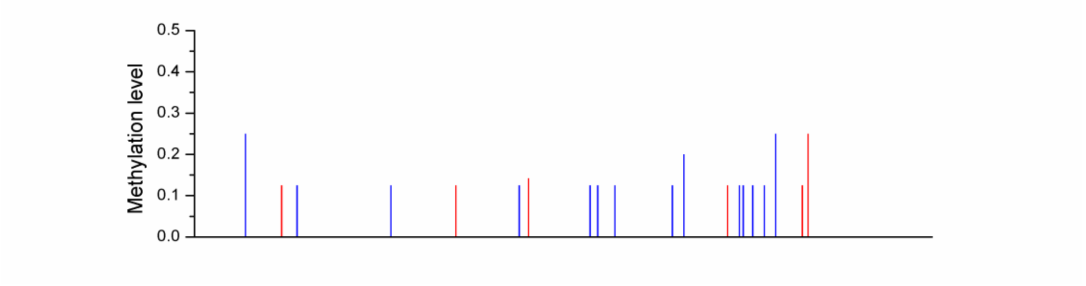

LOC_Os06g16270: heat shock factor binding protein 2 (Hp)

Hp-S

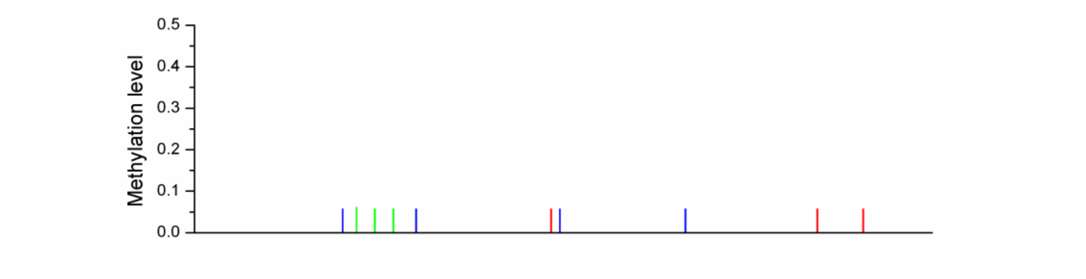

Hp-F

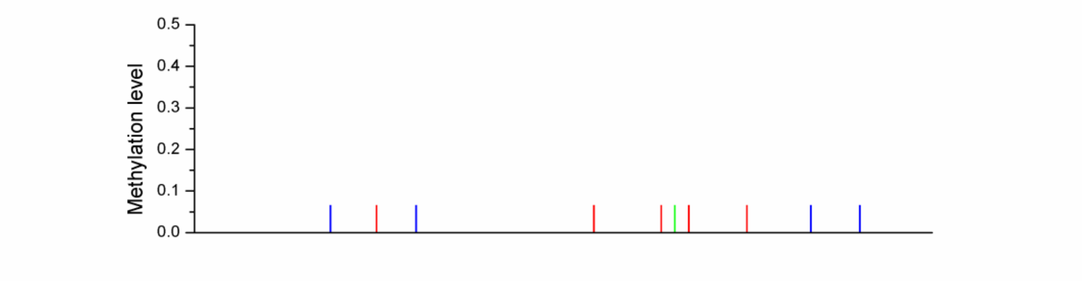

LOC_Os06g23980: putative MADS-box protein AGL16 (MADS)

MADS-S

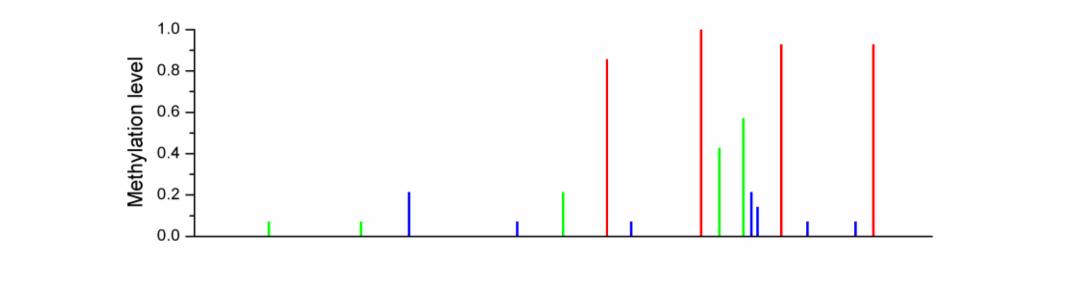

MADS-F

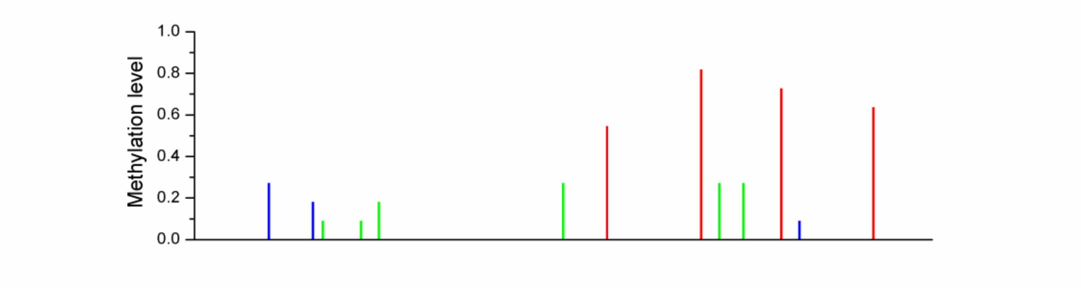

LOC_Os10g10560: invertase/pectin methylesterase inhibitor (MI)

MI-S

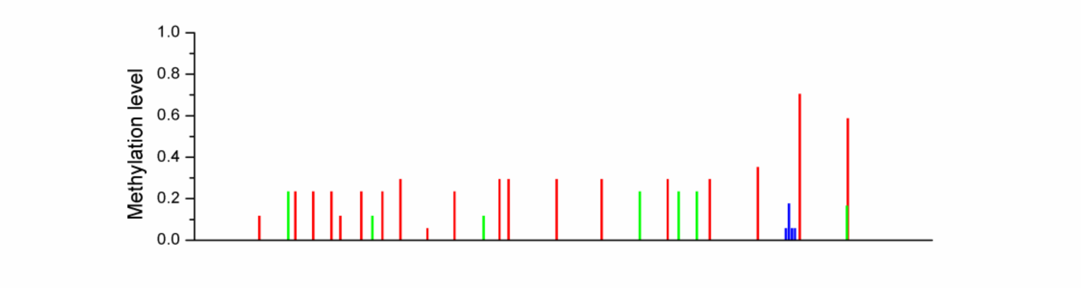

MI-F

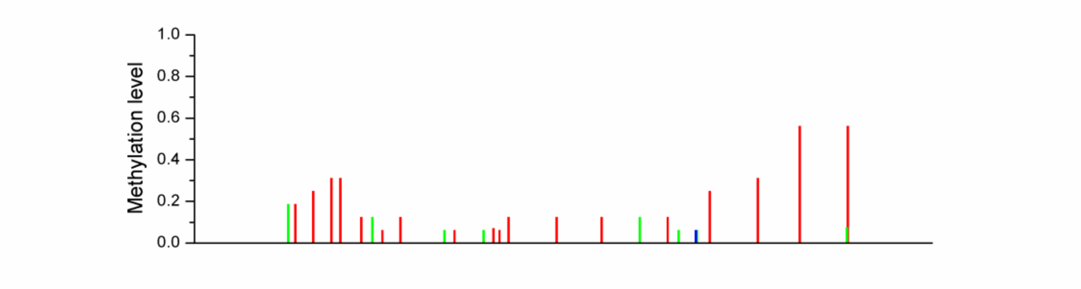

LOC_Os12g07610: MYB family transcription factor (MY)

MY-S

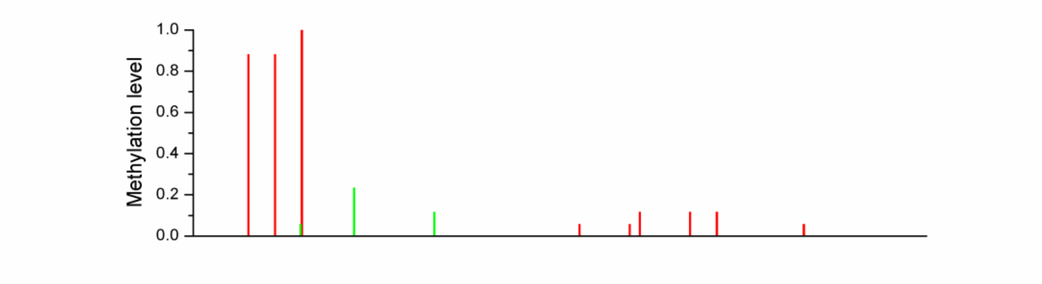

MY-F

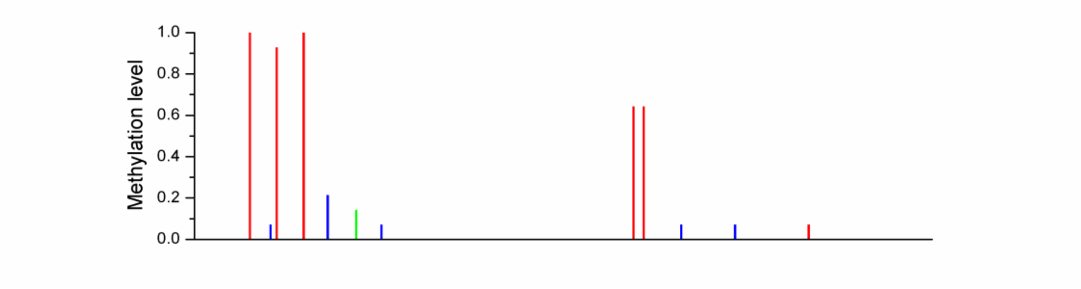

LOC_Os01g13440: pollen allergen Cyn d 23, putative, expressed (PC)

PC-S

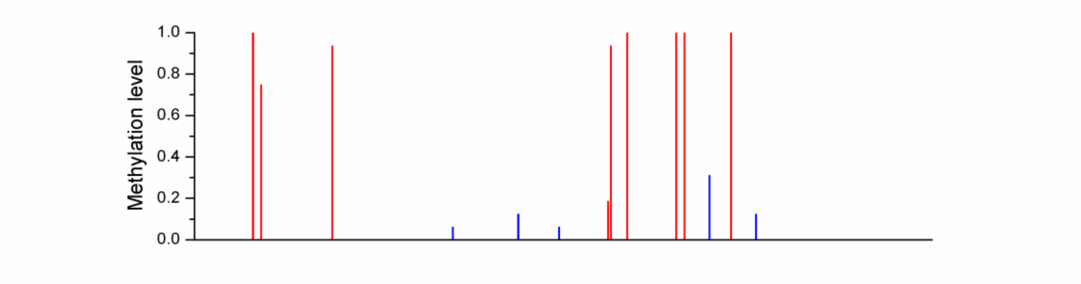

PC-F

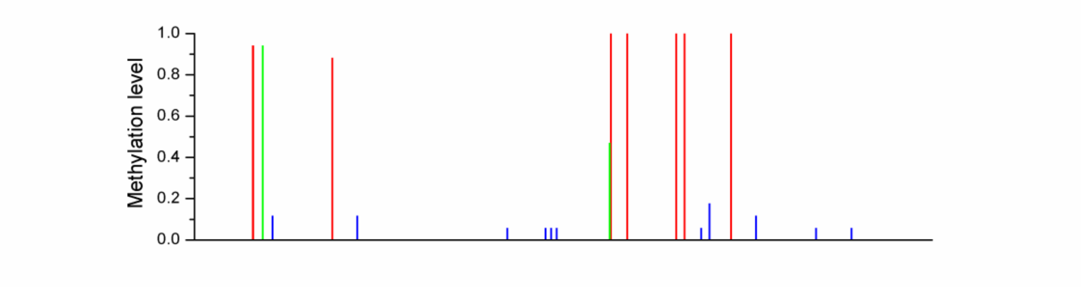

LOC_Os04g37619: Zeaxanthin epoxidase, chloroplastic (OsZEP1)

OsZEP1-S

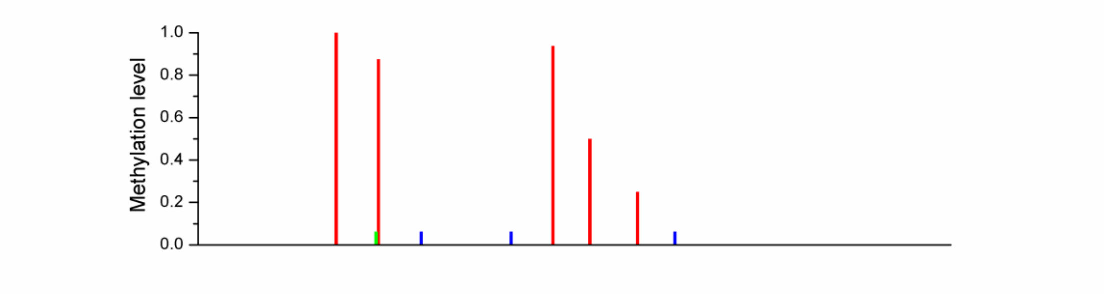

OsZEP1-F

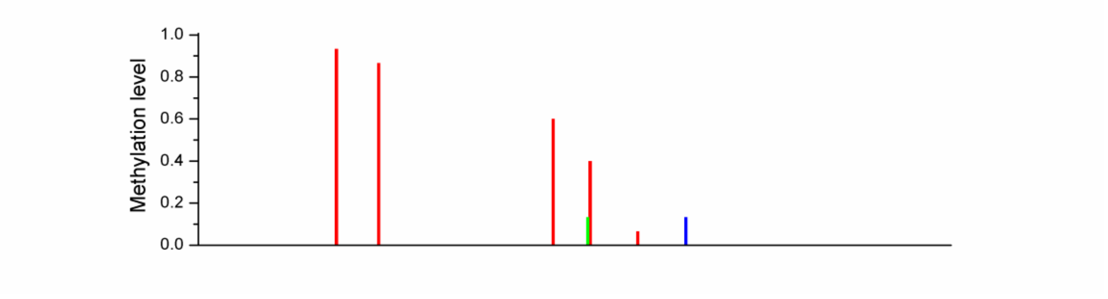

LOC_Os12g08770.1: photosystem I reaction center subunit N (PN)

PN-S

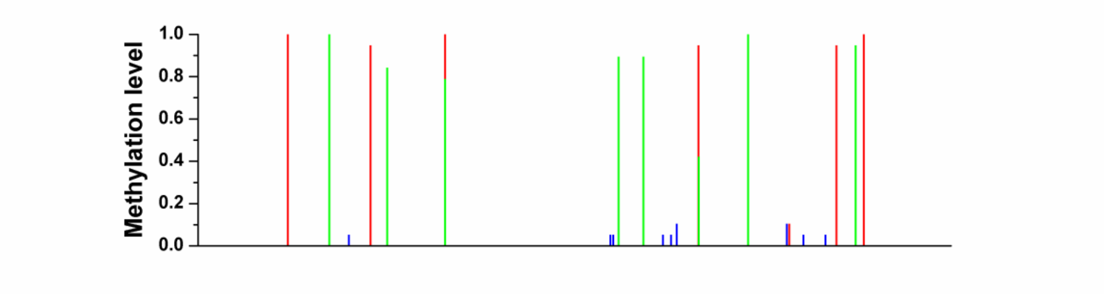

PN-F

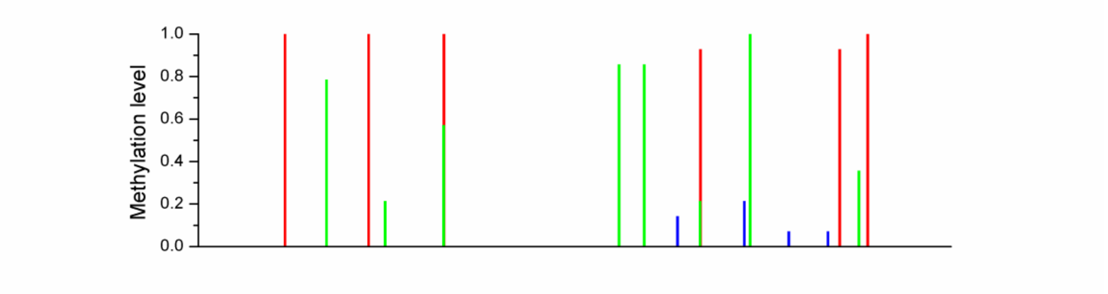

LOC_Os07g17390: PsbP, putative, expressed (PP)

PP-S

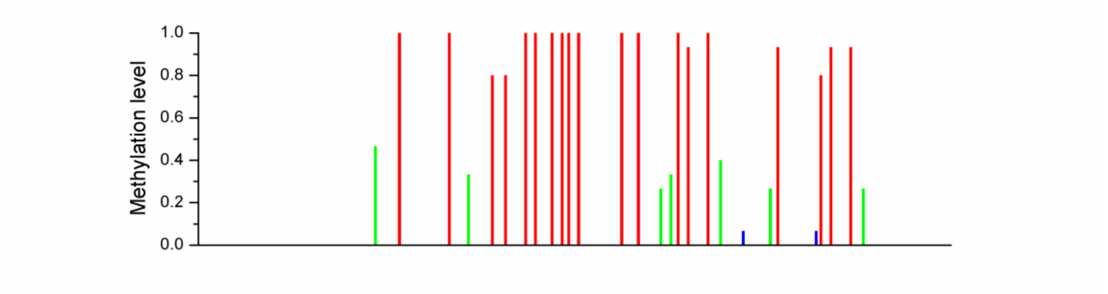

PP-F

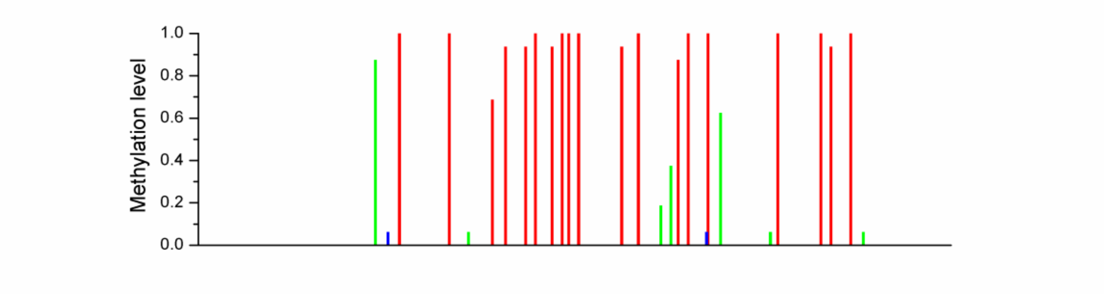

LOC_Os03g27770: heme oxygenase 2, putative, expressed (HO)

HO-S

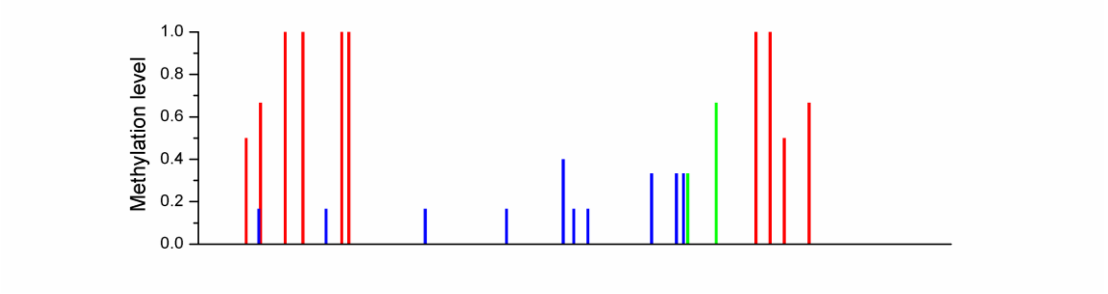

HO-F

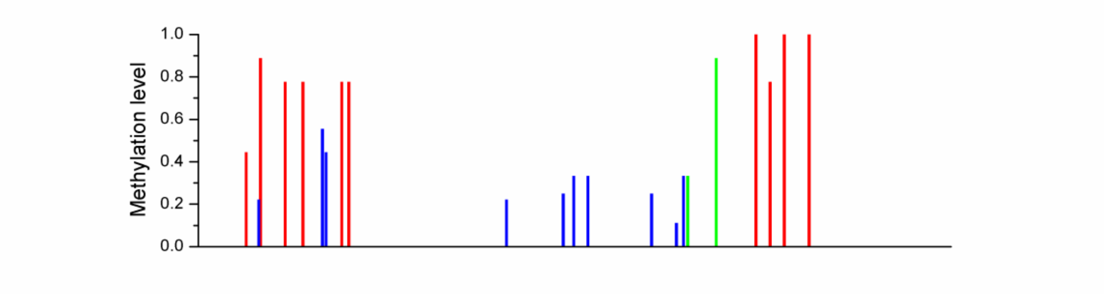

LOC_Os08g38210: transcription factor BIM2, putative, expressed (BM)

BM-S

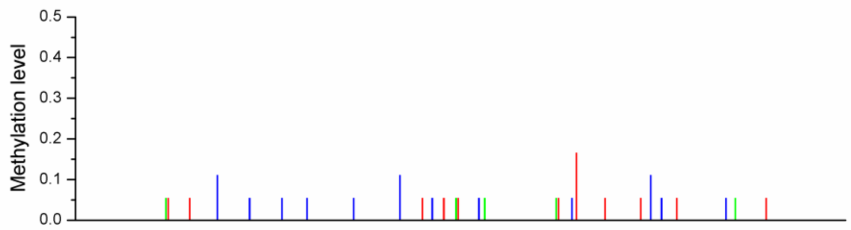

BM-F

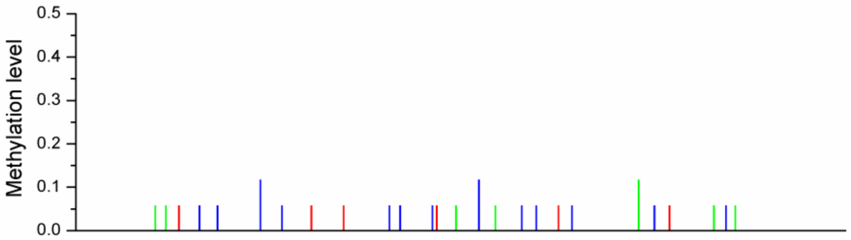

LOC_Os08g41990: aminotransferase, putative, expressed (AM)

AM-S

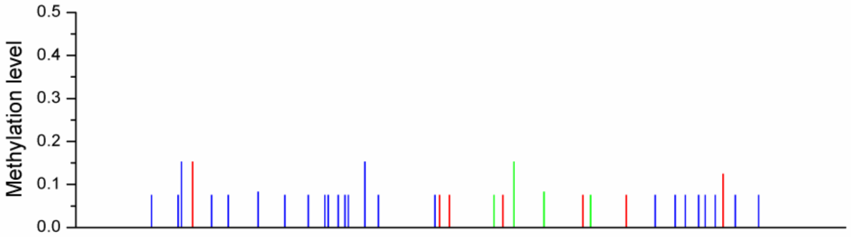

AM-F

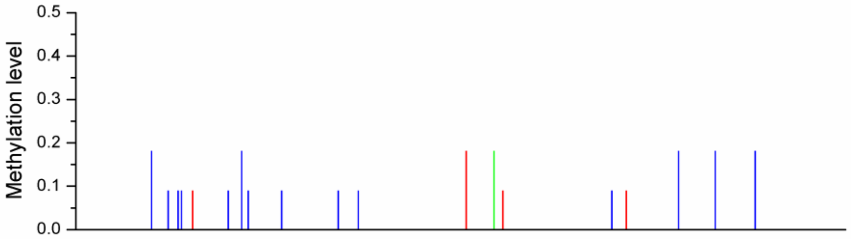

LOC_Os06g40200: calcium-binding mitochondrial carrier (CD)

CD-S

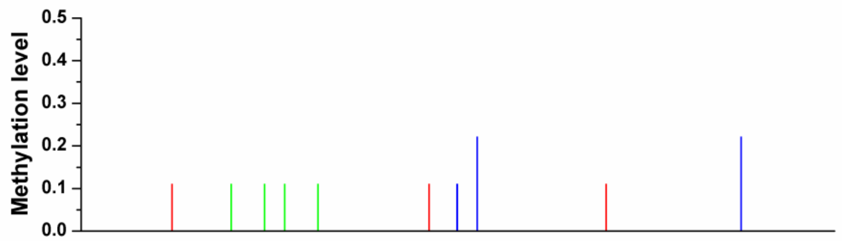

CD-F

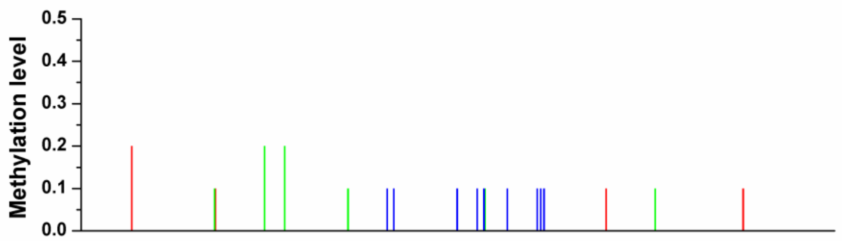

LOC_Os05g12210： chalcone synthase (CS)

CS-S

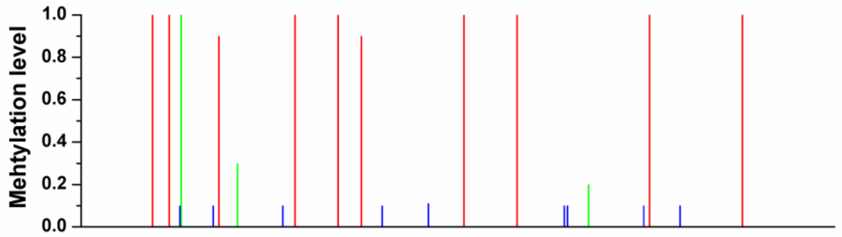

CS-F

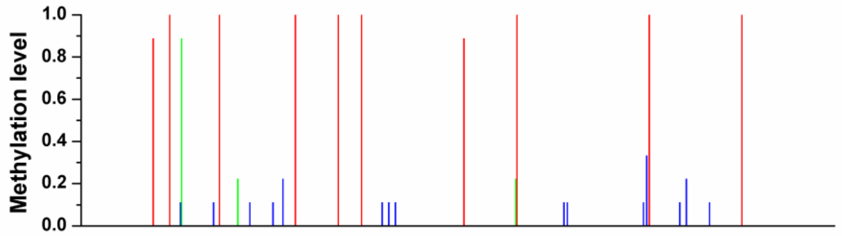

LOC_Os02g17780: ent-kaurene synthase, chloroplast precursor (KS)

KS-S

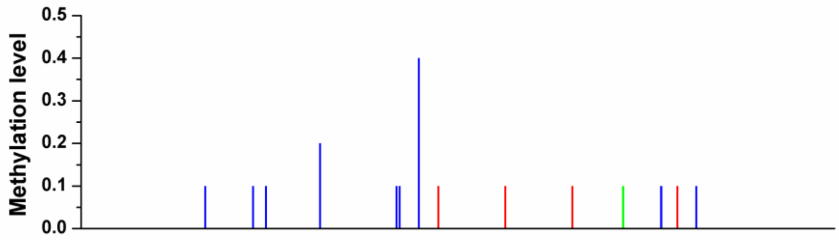

KS-F

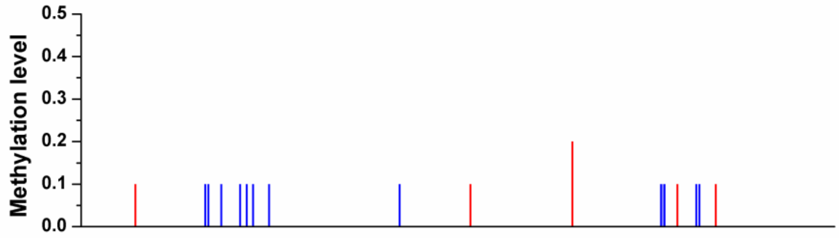

LOC_Os09g38100: phosphate carrier protein, mitochondrial precursor (PH)

PH-S

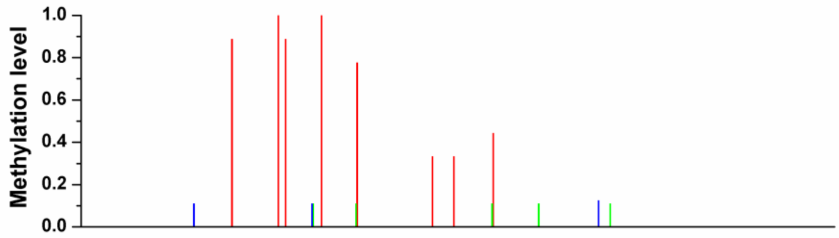

PH-F

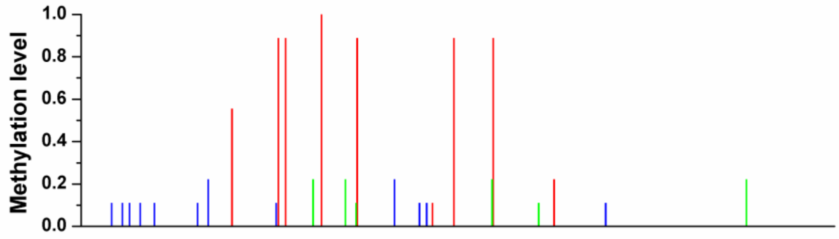

LOC_Os07g02280: Pentatricopeptide (PPR)

PPR-S

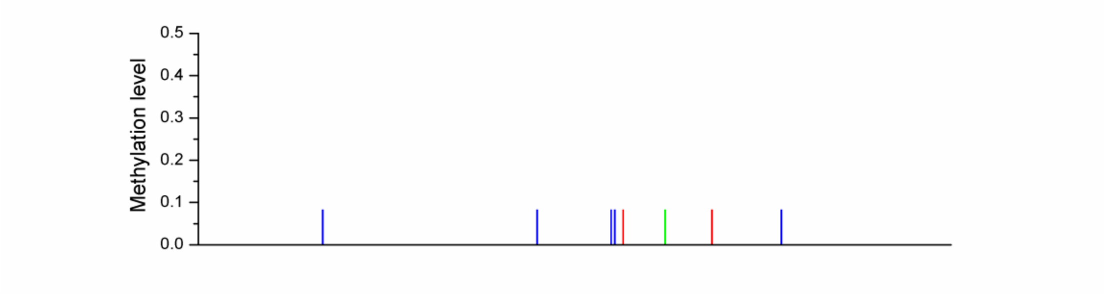

PPR-F

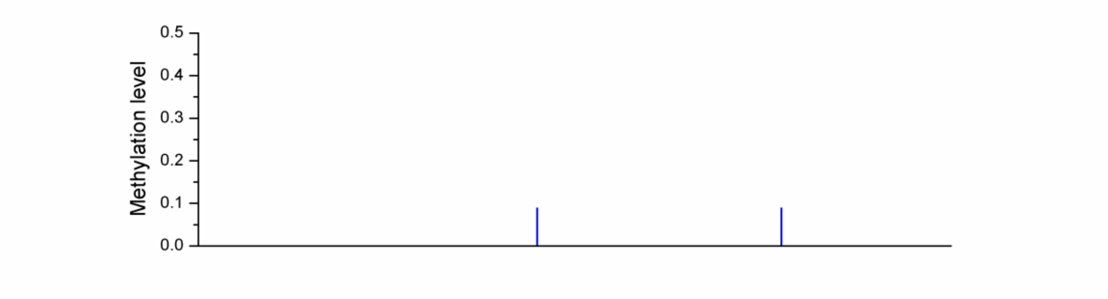

LOC_Os01g67650: gibberellin response modulator-like protein (Grp)

Grp-S

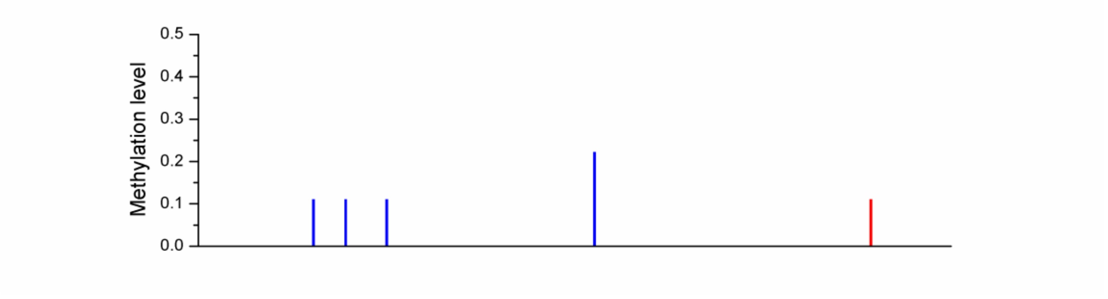

Grp-F

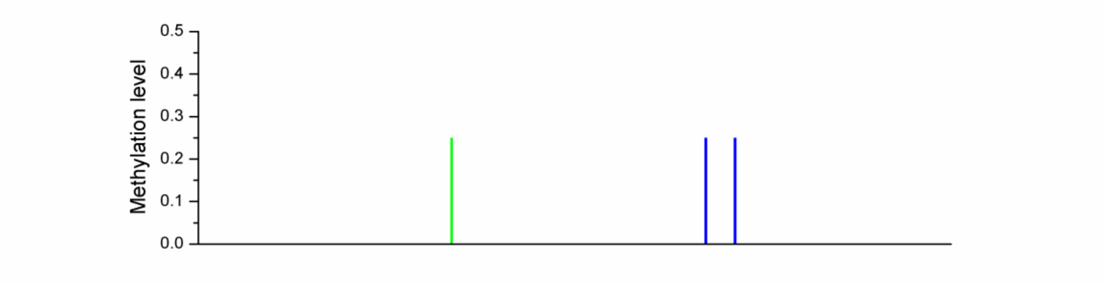

Supplement: Additional file 3: — Bisulfite sequencing of twenty selected DMR-associated genes. The red, green and blue columns in the histograms refer to the collective methylation levels (in percentage) of CG, CHG, and CHH, respectively. [file 12864_2015_1317_MOESM3_ESM.pdf]
