# Supplementary material for: Genome-wide analysis of DNA methylation in photoperiod- and thermo-sensitive male sterile rice Peiai 64S
Source: BMC Genomics. 2015 Feb 19;16(1):102. doi: 10.1186/s12864-015-1317-7 (PMC4367915; doi:10.1186/s12864-015-1317-7)
Supplement: Additional file 6: — Pathways related to the sterility-fertility transition in PA64S. [file 12864_2015_1317_MOESM6_ESM.pdf]

Additional file 5 Pathways related to sterility-fertility transition in PA64S.

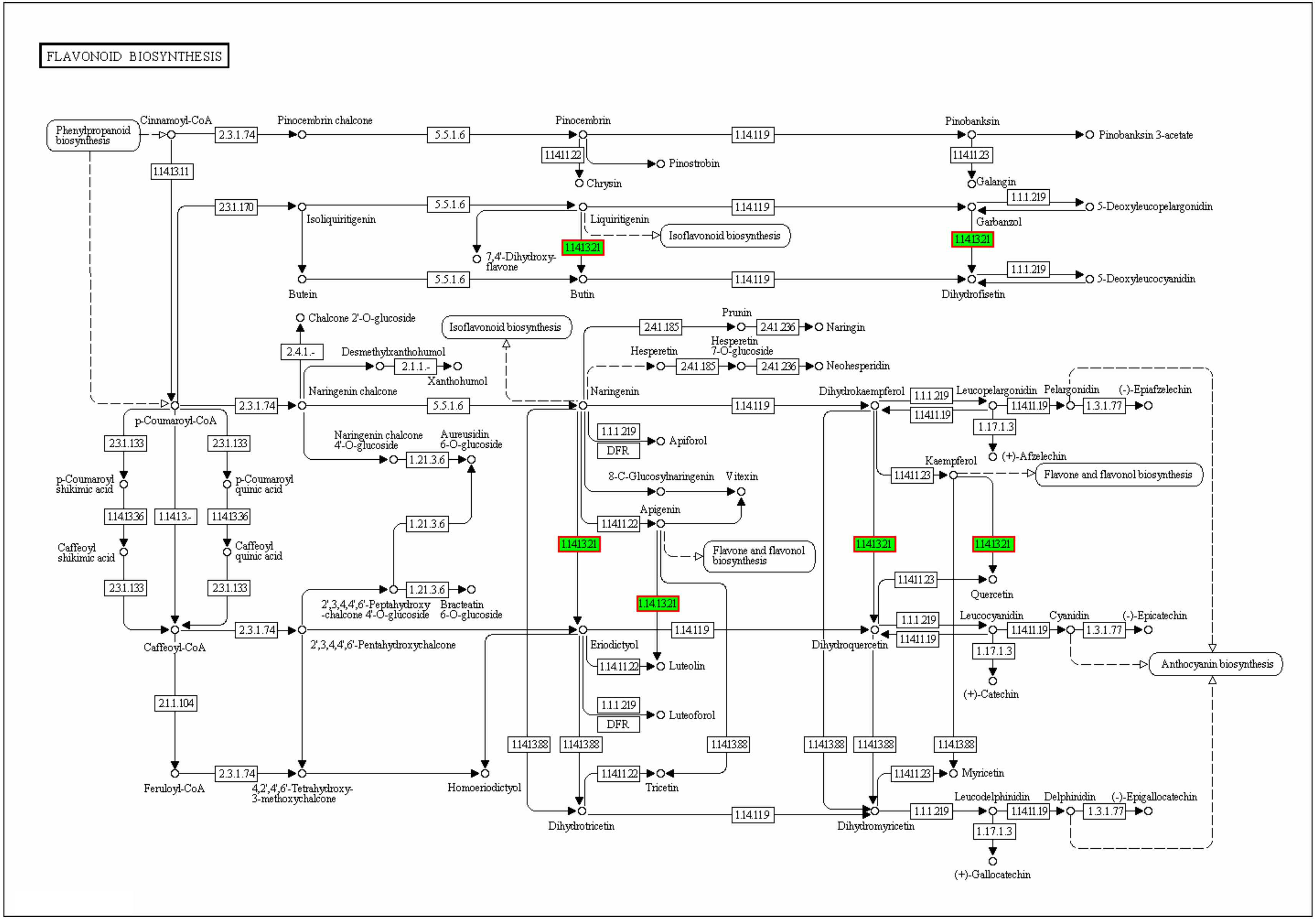

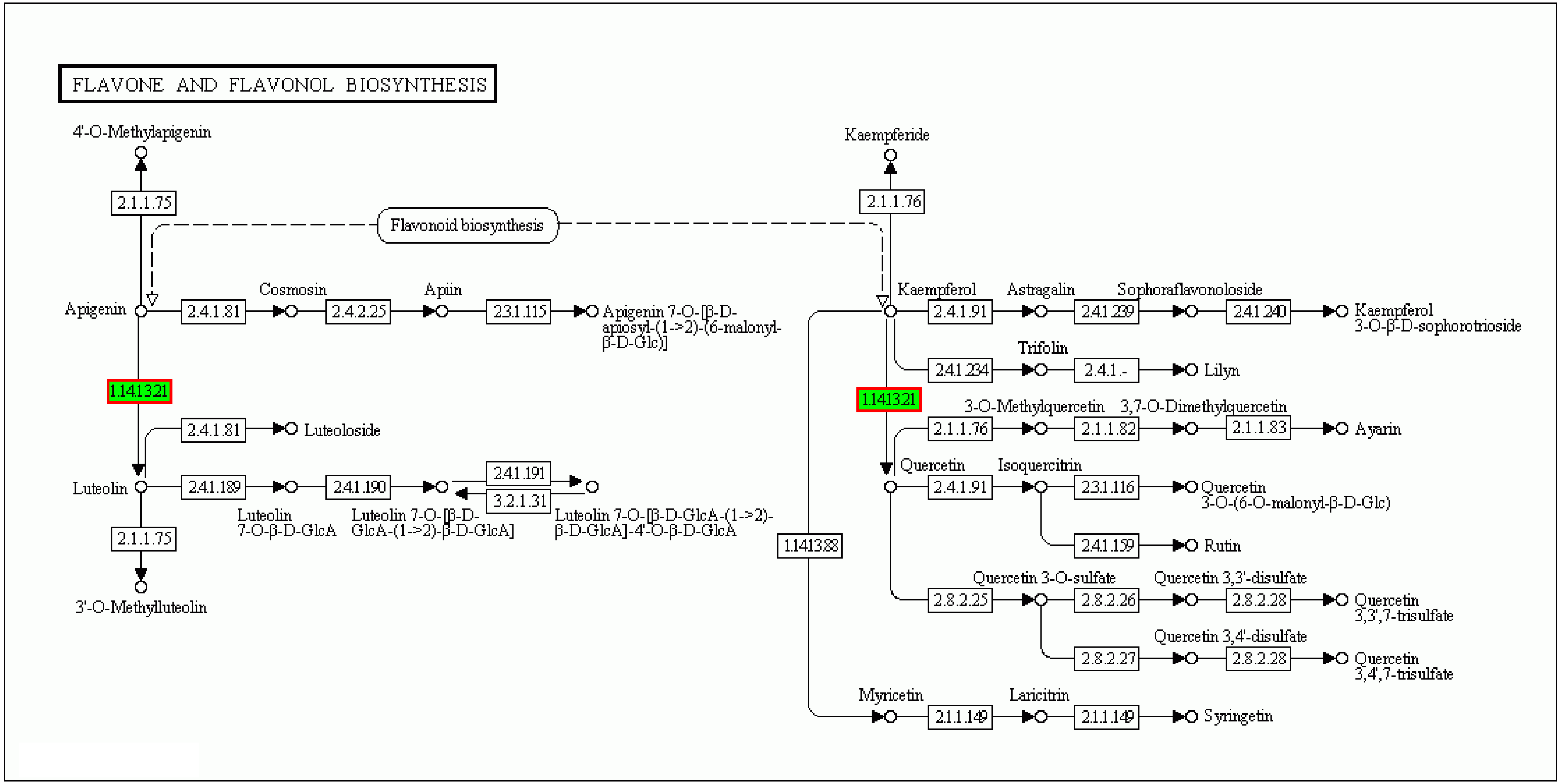
FLAVONE AND FLAVONOL BIOSYNTHESIS
4'-O-Methylapigenin
2.1.1.75
Flavonoid biosynthesis
Kaempferide
2.1.1.76
Apigenin
2.4.1.81
Cosmosin
2.4.2.25
Apiin
2.3.1.115
Apigenin 7-O-[β-D-apiosyl-(1->2)-(6-malonyl-β-D-Glc)]
1.14.13.21
2.4.1.81
Luteoloside
Luteolin
2.4.1.189
2.4.1.190
2.4.1.191
3.2.1.31
Luteolin 7-O-β-D-GlcA
Luteolin 7-O-[β-D-GlcA-(1->2)-β-D-GlcA]
Luteolin 7-O-[β-D-GlcA-(1->2)-β-D-GlcA]-4'-O-β-D-GlcA
2.1.1.75
3'-O-Methylluteolin
Kaempferol
2.4.1.91
Astragalin
2.4.1.239
Sophoraflavonoloside
2.4.1.240
Kaempferol 3-O-β-D-sophorotrioside
2.4.1.234
Trifolin
2.4.1.-
Lilyn
1.14.13.21
3-O-Methylquercetin
3,7-O-Dimethylquercetin
2.1.1.76
2.1.1.82
2.1.1.83
Ayarin
Quercetin
Isoquercitrin
2.4.1.91
2.3.1.116
Quercetin 3-O-(6-O-malonyl-β-D-Glc)
1.14.13.88
2.4.1.159
Rutin
Quercetin 3-O-sulfate
Quercetin 3,3'-disulfate
2.8.2.25
2.8.2.26
2.8.2.28
Quercetin 3,3',7-trisulfate
Quercetin 3,4'-disulfate
2.8.2.27
2.8.2.28
Quercetin 3,4',7-trisulfate
Myricetin
Laricitrin
2.1.1.149
2.1.1.149
Syringetin

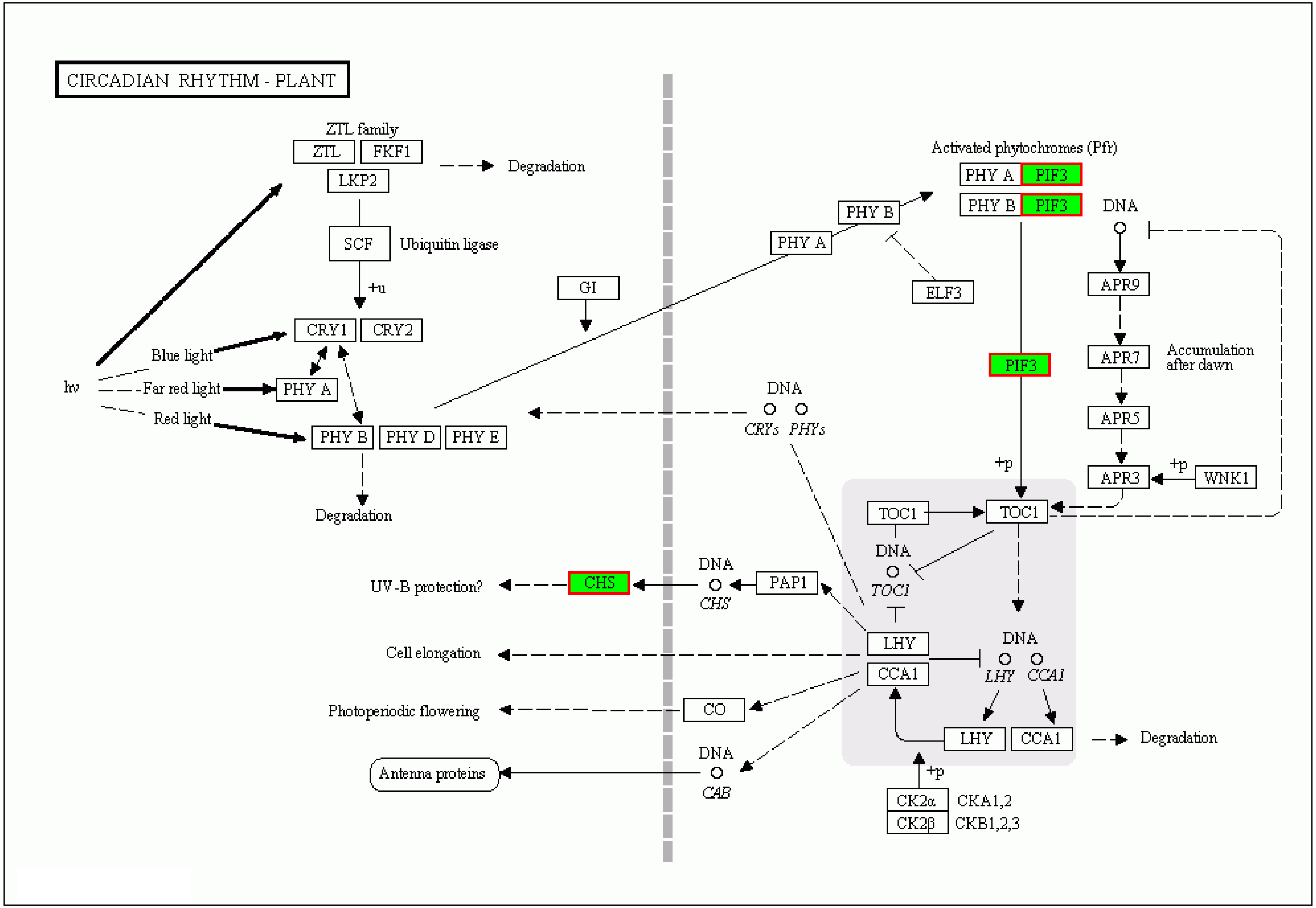

CIRCADIAN RHYTHM - PLANT
ZTL family
ZTL
FKF1
LKP2
Degradation
SCF
Ubiquitin ligase
+u
CRY1
CRY2
GI
hν
Blue light
Far red light
Red light
PHY A
PHY B
PHY D
PHY E
Degradation
DNA
CRYs
PHYs
Activated phytochromes (Pfr)
PHY A
PIF3
PHY B
PIF3
PHY B
PHY A
ELF3
DNA
APR9
APR7
Accumulation after dawn
APR5
APR3
+p
WNK1
PIF3
+p
TOC1
TOC1
DNA
TOC1
UV-B protection?
CHS
DNA
CHS
PAP1
LHY
CCA1
DNA
LHY
CCA1
Cell elongation
Photoperiodic flowering
CO
DNA
CAB
Antenna proteins
LHY
CCA1
Degradation
+p
CK2α
CKA1,2
CK2β
CKB1,2,3

PHOTOSYNTHESIS

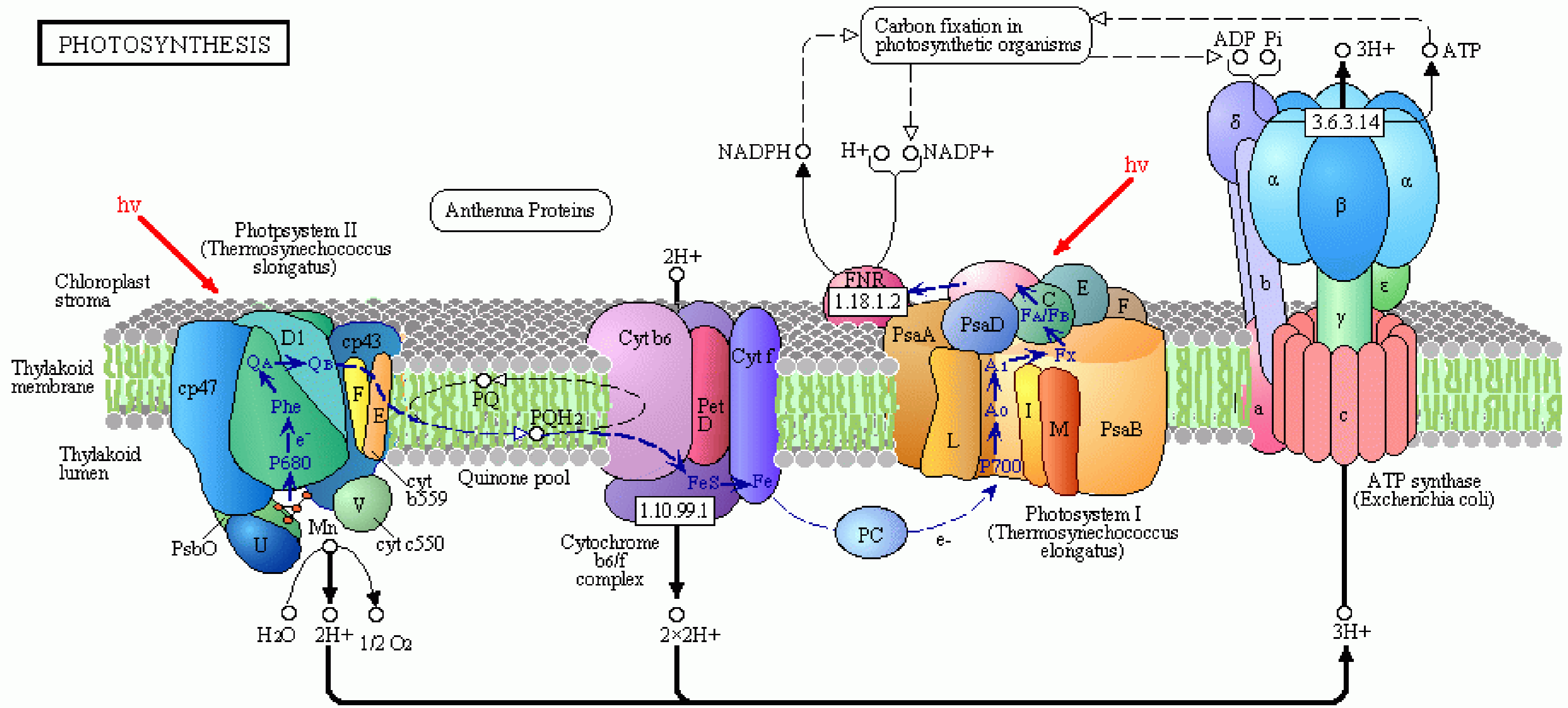

Photosystem II

D1	D2	cp43	cp47	cyt b559	
PsbA	PsbD	PsbC	PsbB	PsbE	PsbF

						MSP	OEC
PsbL	PsbJ	PsbK	PsbM	PsbH	PsbI	PsbO	PsbP
PsbQ	PsbR	PsbS	PsbT	PsbU	PsbV	PsbW	PsbX
PsbY	PsbZ	Psb27	Psb28	Psb28-2			

Photosystem I

PsaA	PsaB	PsaC	PsaD	PsaE	PsaF	PsaG	PsaH
PsaI	PsaJ	PsaK	PsaL	PsaM	PsaN	PsbX	

Cytochrome b6/f complex

PetB	PetD	PetA	PetC	PetL	PetM	PetN	PetG

Photosynthethic electron transport

PC	Fd	FNR	cyt c6
PetE	PetF	PetH	PetJ

F-type ATPase

beta	alpha	gamma	delta	epsilon	c	a	b

OXIDATIVE PHOSPHORYLATION

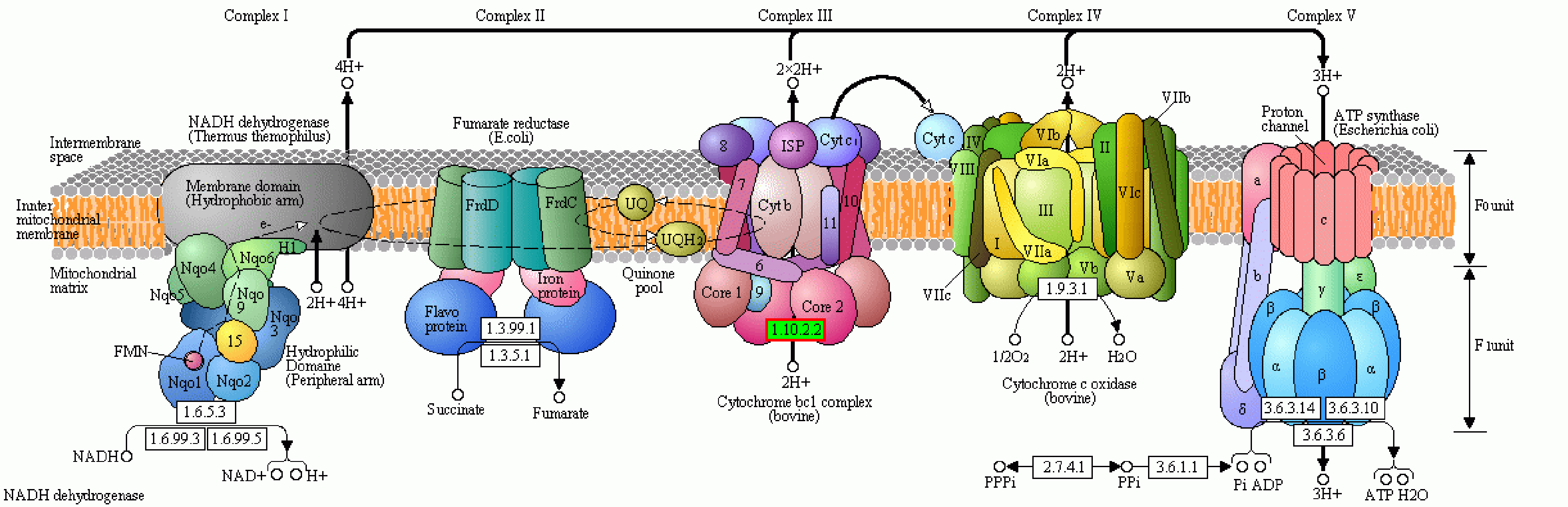

NADH dehydrogenase

E: ND1, ND2, ND3, ND4, ND4L, ND5, ND6

E: Ndufs1, Ndufs2, Ndufs3, Ndufs4, Ndufs5, Ndufs6, Ndufs7, Ndufs8, Ndufv1, Ndufv2, Ndufv3

B/A: NuoA, NuoB, NuoC, NuoD, NuoE, NuoF, NuoG, NuoH, NuoI, NuoJ, NuoK, NuoL, NuoM, NuoN

B/A: NdhC, NdhK, NdhJ, NdhH, NdhA, NdhI, NdhG, NdhE, NdhF, NdhD, NdhB, NdhL, NdhM, NdhN, HoxE, HoxF, HoxU

E: Ndufa1, Ndufa2, Ndufa3, Ndufa4, Ndufa5, Ndufa6, Ndufa7, Ndufa8, Ndufa9, Ndufa10, Ndufab1, Ndufa11

E: Ndufb1, Ndufb2, Ndufb3, Ndufb4, Ndufb5, Ndufb6, Ndufb7, Ndufb8, Ndufb9, Ndufb10, Ndufc1, Ndufc2

Succinate dehydrogenase / Fumarate reductase

E: SDHC, SDHD, SDHA, SDHB

B/A: SdhC, SdhD, SdhA, SdhB
FrdA, FrdB, FrdC, FrdD

Cytochrome c reductase

E/B/A: ISP, Cyt b, Cyt 1

E: COR1, QCR2, QCR6, QCR7, QCR8, QCR9, QCR10

Cytochrome c oxidase

E: COX10, COX3, COX1, COX2, COX4, COX5A, COX5B, COX6A, COX6B, COX6C, COX7A, COX7B, COX7C, COX8, COX17

E/B/A: COX11, COX15

B/A: CyoE, CyoD, CyoC, CyoB, CyoA
CoxD, CoxC, CoxA, CoxB
QoxD, QoxC, QoxB, QoxA

Cytochrome c oxidase , cbb3-type

B: I, II, IV, III

Cytochrome bd complex

B/A: CydA, CydB

F-type ATPase (Bacteria)

beta, alpha, gamma, delta, epsilon, c, a, b

F-type ATPase (Eukaryotes)

beta, alpha, gamma, OSCP, delta, epsilon, c, a
b, e, f6, f, 8
d, f, h, j, k, g

F-type ATPase (Prokaryotes)

A, B, C, D, E, F, I, K

F-type ATPase (Eukaryotes)

A, B, C, D, E, F, G, H
I, AC39, 54kD, S1, lipid

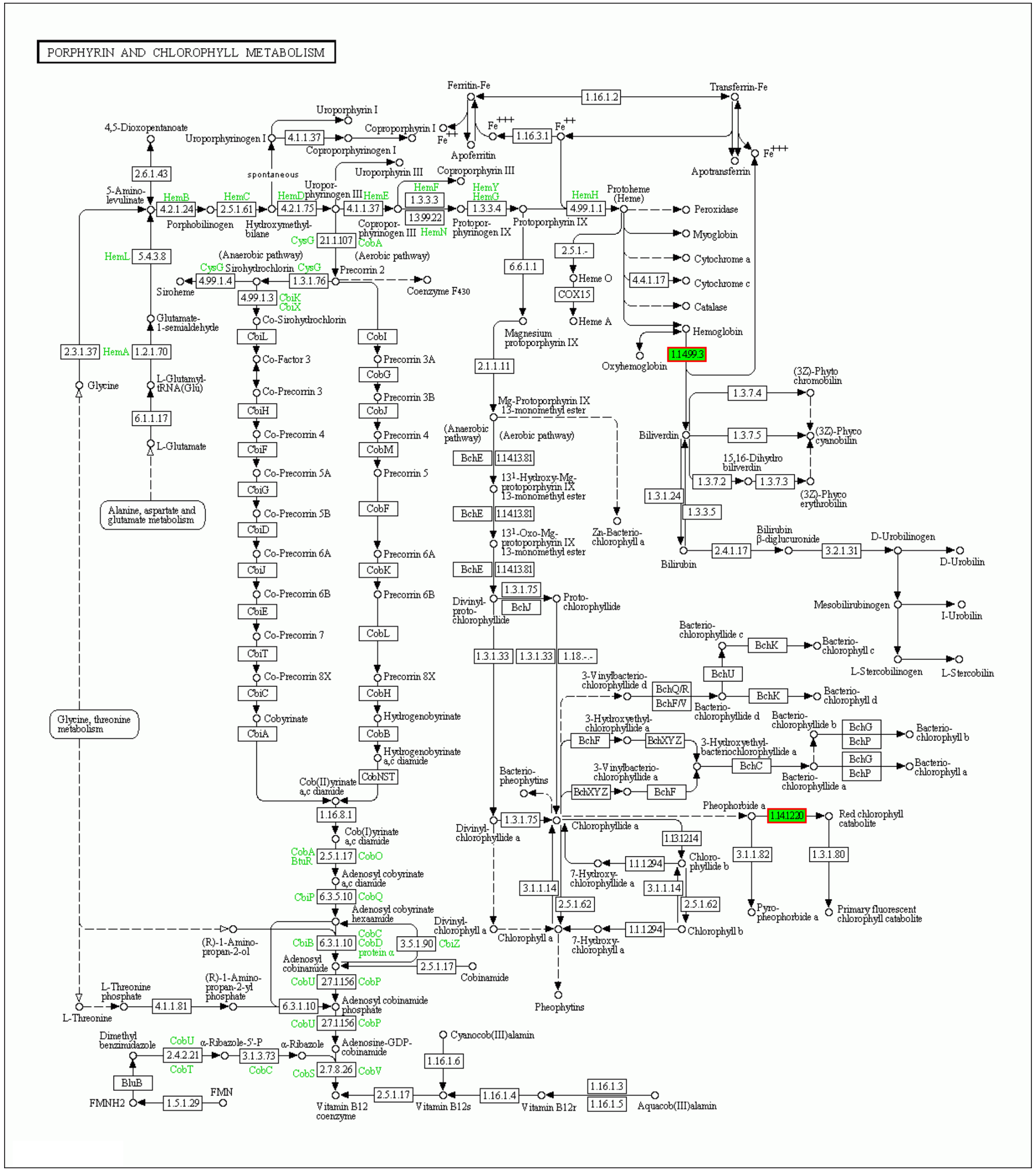
PORPHYRIN AND CHLOROPHYLL METABOLISM
Ferritin-Fe
1.16.1.2
Transferrin-Fe
Uroporphyrin I
Coproporphyrin I
4,5-Dioxopentanoate
Uroporphyrinogen I
4.1.1.37
Coproporphyrinogen I
1.16.3.1
Apoferritin
Apotransferrin
2.6.1.43
spontaneous
Uroporphyrin III
Coproporphyrin III
5-Amino-levulinate
HemB
4.2.1.24
HemC
2.5.1.61
HemD
4.2.1.75
Uropor-phyrinogen III
HemE
4.1.1.37
HemF
1.3.3.3
1.3.99.22
HemN
HemY
HemG
1.3.3.4
HemH
4.99.1.1
Protoheme (Heme)
Peroxidase
Myoglobin
Cytochrome a
Cytochrome c
Catalase
Hemoglobin
Porphobilinogen
Hydroxymethyl-bilane
Copropor-phyrinogen III
Protopor-phyrinogen IX
Protoporphyrin IX
CysG
2.1.1.107
CobA
HemL
5.4.3.8
(Anaerobic pathway)
(Aerobic pathway)
Sirohydrochlorin
4.99.1.4
1.3.1.76
Siroheme
Precorrin 2
Coenzyme F430
2.5.1.-
Heme O
4.4.1.17
COX15
Heme A
6.6.1.1
4.99.1.3
CbiK
CbiX
Co-Sirohydrochlorin
Glutamate-1-semialdehyde
Magnesium protoporphyrin IX
2.3.1.37
HemA
1.2.1.70
CbiL
CobI
Co-Factor 3
Precorrin 3A
2.1.1.11
1.14.99.3
Oxyhemoglobin
Glycine
L-Glutamyl-tRNA(Glu)
Co-Precorrin 3
CobG
Precorrin 3B
CbiH
CobJ
Mg-Protoporphyrin IX 13-monomethyl ester
1.3.7.4
(3Z)-Phyto chromobilin
6.1.1.17
Co-Precorrin 4
Precorrin 4
(Anaerobic pathway)
(Aerobic pathway)
Biliverdin
1.3.7.5
(3Z)-Phyco cyanobilin
L-Glutamate
CbiF
CobM
BchE
1.14.13.81
15,16-Dihydro biliverdin
1.3.7.2
1.3.7.3
Co-Precorrin 5A
Precorrin 5
CbiG
Alanine, aspartate and glutamate metabolism
Co-Precorrin 5B
CobF
1.3.1.24
(3Z)-Phyco erythrobilin
1.3.3.5
CbiD
Co-Precorrin 6A
Precorrin 6A
Zn-Bacterio-chlorophyll a
Bilirubin β-diglucuronide
D-Urobilinogen
2.4.1.17
3.2.1.31
Bilirubin
D-Urobilin
CbiJ
CobK
Co-Precorrin 6B
Precorrin 6B
1.3.1.75
BchJ
Divinyl-proto-chlorophyllide
Proto-chlorophyllide
Mesobilirubinogen
I-Urobilin
CbiE
Co-Precorrin 7
CobL
Bacterio-chlorophyllide c
BchK
Bacterio-chlorophyll c
CbiT
1.3.1.33
1.18.-.-
L-Stercobilinogen
L-Stercobilin
Co-Precorrin 8X
Precorrin 8X
BchU
3-Vinylbacterio-chlorophyllide d
BchQ/R
BchF/V
Bacterio-chlorophyllide d
Bacterio-chlorophyll d
CbiC
CobH
Glycine, threonine metabolism
Cobyrinate
Hydrogenobyrinate
3-Hydroxyethyl-chlorophyllide a
BchF
BchXYZ
Bacterio-chlorophyllide b
BchG
BchP
Bacterio-chlorophyll b
CbiA
CobB
3-Hydroxyethyl-bacteriochlorophyllide a
Hydrogenobyrinate a,c diamide
BchC
Bacterio-chlorophyll a
Bacterio-pheophytins
3-Vinylbacterio-chlorophyllide a
Bacterio-chlorophyllide a
CobNST
Cob(II)yrinate a,c diamide
Pheophorbide a
1.14.12.20
Red chlorophyll catabolite
1.16.8.1
Divinyl-chlorophyllide a
Chlorophyllide a
Cob(I)yrinate a,c diamide
1.13.12.14
CobA
BtuR
2.5.1.17
CobO
3.1.1.82
1.3.1.80
1.1.1.294
Chloro-phyllide b
Adenosyl cobyrinate a,c diamide
7-Hydroxy-chlorophyllide a
3.1.1.14
CbiP
6.3.5.10
CobQ
2.5.1.62
Pyro-pheophorbide a
Primary fluorescent chlorophyll catabolite
Adenosyl cobyrinate hexaamide
Divinyl-chlorophyll a
Chlorophyll a
Chlorophyll b
(R)-1-Amino-propan-2-ol
CbiB
6.3.1.10
CobC
CobD
protein α
3.5.1.90
CbiZ
7-Hydroxy-chlorophyll a
Adenosyl cobinamide
2.5.1.17
Cobinamide
CobU
2.7.1.156
CobP
L-Threonine phosphate
(R)-1-Amino-propan-2-yl phosphate
Pheophytins
L-Threonine
4.1.1.81
6.3.1.10
Adenosyl cobinamide phosphate
Cyanocob(III)alamin
Dimethyl benzimidazole
CobU
α-Ribazole-5'-P
α-Ribazole
CobT
2.4.2.21
3.1.3.73
CobC
Adenosine-GDP-cobinamide
CobS
2.7.8.26
CobV
1.16.1.6
BluB
1.16.1.3
FMN
1.5.1.29
FMNH2
2.5.1.17
1.16.1.4
1.16.1.5
Vitamin B12 coenzyme
Vitamin B12s
Vitamin B12r
Aquacob(III)alamin
