# Supplementary material for: Genome-wide analysis of DNA methylation in photoperiod- and thermo-sensitive male sterile rice Peiai 64S
Source: BMC Genomics. 2015 Feb 19;16(1):102. doi: 10.1186/s12864-015-1317-7 (PMC4367915; doi:10.1186/s12864-015-1317-7)
Supplement: Additional file 7: — Primers for bisulfite sequencing and qRT-PCR. [file 12864_2015_1317_MOESM7_ESM.pdf]

Additional file 7 Primers for bisulfite sequencing and qRT-PCR.

Primer name	Primer sequences (5'–3')
**Bisulfite sequencing**	
AT-F	GGTATTTATTATTTTTTAGTTTTTTTTATT
AT-R	AATTCAAACACCAATTACAACTTC
DJ-F	ATGTTTAAAGAAAAGAGTATTATTATTTAA
DJ-R	AAACAAAAAACAAATCTAAAAAAAC
H2A-F	ATGATATTTTATTTTTTTTATGTATTGTAA
H2A-R	AACTAACCTAAAATTTTCTTTTT
HSP-F	GGTATTTTTATTAAGGTATATTTTTTTT
HSP-R	CAAATACAAAAACTACCCACAACC
MADS-F	GGTTGTTTTGAAGAATGATTTTTATAG
MADS-R	ATTTTTAACAAACAAAAACTCCTAC
MI-F	ATTTTTTTTAGAGAGTTAGATGAA
MI-R	AAAATTATAAAAAAATATTTTTATCC
MY-F	GGAAATAATGTAGATTTTTTAAGTTATTT
MY-R	ATAAAATTAAACTCTCTTTTTCTTTTAAAA
PC-F	ATGTAATTATATGTTTTGTTAGGGG
PC-R	ACATCAAAAACCATCATCCTAAAAC
OsZEP1-F	TTGTTTTGAATATTTTGGTAAAAAT
OsZEP1-R	ACCAAATTCTCACCATCTTCTATAC
PN-F	TAATGGATATGATAGTTTTGAATATGATT
PN-R	AACCCTAAATTACAAAAACACAAAC
PP-F	TAAGAGTAAATTAAATAAAAATTGGTTGTA
PP-R	AAACCACTACAACTACCCAAAATAC
HO-F	TGGGTGTTGGGAATTTATTATTTA
HO-R	ATATTTACAACCCCTTATTTCCAAC
BM-F	TGTGTTTTGTGTTGTTTGTTTATT
BM-R	AAATTAAAATACTCCACCTTTCAAC
AM-F	AAATTGTGGGATTTATTGATATGTG
AM-R	AAAAAAACCTCTCCTTCCCATAA
CD-F	AGGGGAGTTGAATTGGGTAAT
CD-R	CCAAACTTTACTAAAATTATTTCTAACAAC
CS-F	TTTAGGGTATGTTTTATTTGGAAGTAT
CS-R	AAACAAAAAATAAAAAAACAAAACAC
KS-F	GAGTTAATTTATTTTTTAAGGTTGG
KS-R	CTACTCTACTCTCTATTCTTCTCCTCTAAT
PH-F	ATTTTTTTATTTTTATATTTTAATATAGTG
PH-R	AATCTAAAATTCCTACTACTTTTTC
PPR-F	GGTTTAATAGTTTATAAGTGTTTTTTTT
PPR-R	TAAATACACCATACTACTAACCTCC
Grp-F	ATATTGGATGAGTGAAATTTATTTTATTAT
Grp-R	AAACTATCTAACATCTCAAACTAACC

**Primer name**	**ID**	**Primer sequences (5’–3’)**	**Purpose**
**qRT-PCR**			
H2A-F	LOC_Os03g51200	TACCTCTCCGCCGTCCTC	
H2A-R		TCAGCTCCTCGTCGTTGC	
HSP-F	LOC_Os06g16270	GATCAGGACTCGGATGGC	
HSP-R		TGAAGTGATTGCATGAAATTCG	
HO-F	LOC_Os03g27770	ACCCTCAAGATGGACGATACGG	Methylated genes
HO-R		CGGTGGACTCGGCTACGAT	
BM-F	LOC_Os08g38210	AATAATTCTGACCTTGCCATCG	
BM-R		AGTAGATCTCTTCATAGCCCTC	
OsSPL8-F	LOC_Os04g56170	GTCGTCTGGCTCGTGCTTATTC	
OsSPL8-R		TGTGTTGGTGATGCTGCTGATG	
OsMS2-F	LOC_Os08g44360	ACATCATCTACGACGACCTG	
OsMS2-R		TCCAGCGACACATCATACC	
OsBRI1-F		AGCCTCAACTACATCAATGGGTC	
OsBRI1-R		GGTATCTCGCCCTCCAGCT	BR signaling pathway
OsBZR1-F		ACCTACCGCAAGGGATGT	
OsBZR1-R		CTCAGCAGCTGCGTTGAC	
OsBAK1-A-F		GAGTTGATCTTGGGAATGCTGC	
OsBAK1-A-R		CACTAGGTATCGTTCCGCTTATGTT	
OsBAK1-B-F		TCTTTGATGTGCCTGCTGA	
OsBAK1-B-R		TTTATTGCTGAAGGTATCTGTTG	
actin1-F		AGCAACTGGGATGATATGGA	
actin1-R		CAGGGCGATGTAGGAAAGC	
